# Trophic factor BDNF inhibits GABAergic signaling by facilitating dendritic enrichment of SUMO E3 ligase PIAS3 and altering gephyrin scaffold

**DOI:** 10.1016/j.jbc.2022.101840

**Published:** 2022-03-17

**Authors:** Zahra S. Thirouin, Marta Figueiredo, Mohammad Hleihil, Raminder Gill, Giovanna Bosshard, R Anne McKinney, Shiva K. Tyagarajan

**Affiliations:** 1 Institute of Pharmacology and Toxicology, University of Zürich, Zürich, Switzerland; 2 Department of Pharmacology and Therapeutics, McGill University, Montreal, Canada

**Keywords:** sumoylation, ischemia, synaptic plasticity, posttranslational modification, neurotrophin, BDNF, brain-derived neurotrophic factor, IP, immunoprecipitation, mIPSCs, miniature inhibitory postsynaptic currents, OGD, oxygen–glucose deprivation, PIAS, protein inhibitor of activated STAT, SUMO, small ubiquitin-like modifier, TrkB, tropomyosin-related kinase B, WB, Western blot

## Abstract

Posttranslational addition of a small ubiquitin-like modifier (SUMO) moiety (SUMOylation) has been implicated in pathologies such as brain ischemia, diabetic peripheral neuropathy, and neurodegeneration. However, nuclear enrichment of SUMO pathway proteins has made it difficult to ascertain how ion channels, proteins that are typically localized to and function at the plasma membrane, and mitochondria are SUMOylated. Here, we report that the trophic factor, brain-derived neurotrophic factor (BDNF) regulates SUMO proteins both spatially and temporally in neurons. We show that BDNF signaling *via* the receptor tropomyosin-related kinase B facilitates nuclear exodus of SUMO proteins and subsequent enrichment within dendrites. Of the various SUMO E3 ligases, we found that PIAS-3 dendrite enrichment in response to BDNF signaling specifically modulates subsequent ERK1/2 kinase pathway signaling. In addition, we found the PIAS-3 RING and Ser/Thr domains, albeit in opposing manners, functionally inhibit GABA-mediated inhibition. Finally, using oxygen–glucose deprivation as an *in vitro* model for ischemia, we show that BDNF–tropomyosin-related kinase B signaling negatively impairs clustering of the main scaffolding protein at GABAergic postsynapse, gephyrin, whereby reducing GABAergic neurotransmission postischemia. SUMOylation-defective gephyrin K148R/K724R mutant transgene expression reversed these ischemia-induced changes in gephyrin cluster density. Taken together, these data suggest that BDNF signaling facilitates the temporal relocation of nuclear-enriched SUMO proteins to dendrites to influence postsynaptic protein SUMOylation.

The family of small ubiquitin-like modifier (SUMO) proteins initially identified in *Saccharomyces cerevisiae* is now known to be expressed in all eukaryotes (1). SUMO conjugation on substrate proteins occurs over three-step process involving ATP and SUMO-specific enzymes. While the SUMO-1, -2, -3 proteins are expressed from three different genes in humans, only one E2 conjugating enzyme, Ubc9, has been described in eukaryotes (2). E3 ligases trigger SUMO conjugation on substrates by recruitment of Ubc9. They consist of two major classes, namely HECT-domain and RING-domain type ligases. The RING-type ligases bind both substrate and Ubc9 (3). Protein inhibitor of activated STAT (PIAS) family of RING-type SUMO E3 ligase are well described in literature for their SUMO-conjugating role in eukaryotes (4, 5, 6). The initial link between SUMOylation and nucleocytoplasmic transport was established when the import factor RanGAP1 SUMOylation was shown to localize it to the nuclear pore (7). Subsequently, numerous independent reports have shown that several cellular proteins alter their nucleocytoplasmic distribution and function upon SUMOylation (8, 9). Although most SUMO conjugates described in the literature are localized within the nucleus, SUMO modification can also occur outside the nucleus as SUMOylation of membrane receptors (GluK2 and Kv2.1; (10, 11)); cytosolic proteins (CASK), syntaxin1 and gephyrin (12, 13); and metabolic enzymes localized within the cytoplasm have been reported (14). Even though the controversy surrounding the intracellular site for SUMO conjugation has dissipated, our understanding about the occurrence rate of protein SUMOylation and its upstream signal(s) remains limited.

In neurons, SUMO conjugation of cytoplasmic and membrane proteins influences cell physiology by allowing rapid adaptations to shifts in cellular metabolism *via* intermolecular and intramolecular interaction (13, 14). Therefore, SUMOylation of synaptic proteins has emerged as a critical regulator of synaptic plasticity (15). For example, SUMOylation has also been shown to contribute to the GABAergic postsynapse organization through both SUMO-1 and SUMO-2 conjugation on gephyrin, the main inhibitory scaffolding protein (13). In the same study, it was reported that PIAS-3 and SENP-2 modulate gephyrin SUMOylation levels (at K148 and K724 residues) downstream of α2 GABA_A_Rs to facilitate scaffolding at inhibitory postsynaptic membrane (13).

While SUMO substrates and the functional consequences of SUMOylation are becoming clear, the upstream signaling that facilitates SUMO conjugation onto substrates remains less well understood. It has been reported that under conditions of cellular stress, protein SUMOylation increases (16, 17), and after ischemia, brain-derived neurotrophic factor (BDNF) levels transiently increase (18). Although a functional link between the BDNF and SUMO pathway has not been established in literature, acute application of BDNF has been reported to weaken GABAergic transmission (19, 20) and GABA_A_R surface expression in hippocampal primary (21). BDNF signaling has also been linked to ubiquitin-mediated GABA_A_ receptor internalization and degradation in neurons (21). Furthermore, it is reported that GABA_A_Rs are rapidly depleted from synapses *via* AP2-dependent endocytosis following ischemia (22). At the molecular level, BDNF activation of its high affinity receptor, tropomyosin-related kinase B (TrkB) receptor, could influence SUMOylation of the main scaffolding protein gephyrin, whereby contributing to reduced cell surface expression of GABA_A_R and gephyrin clustering.

In the current study, we report that BDNF signaling regulates nucleocytoplasmic transport of SUMO-1, SUMO-2/3, and PIAS-3 proteins in neuronal cells. Specifically, PIAS-3 is the only member of the E3 ligase family whose cytoplasmic localization in neuronal cells are reversibly affected by the duration of TrkB activation. At a mechanistic level, we report functional uncoupling between PIAS-3 RING-domain and C-terminus S/T domain influences GABAergic neurotransmission changes. We identify ERK1/2 kinase pathway as downstream effector of PIAS-3 nuclear localization and function. Finally, we uncover that ischemia in hippocampal slices induces loss of gephyrin clusters and GABAergic synaptic transmission. Moreover, this gephyrin cluster loss can be rescued by transgenic expression of SUMO-defective gephyrin K148R/K724R mutant or BDNF scavenging.

## Results

### Acute BDNF treatment alters subcellular localization of SUMO pathway proteins

**Figure 1:**
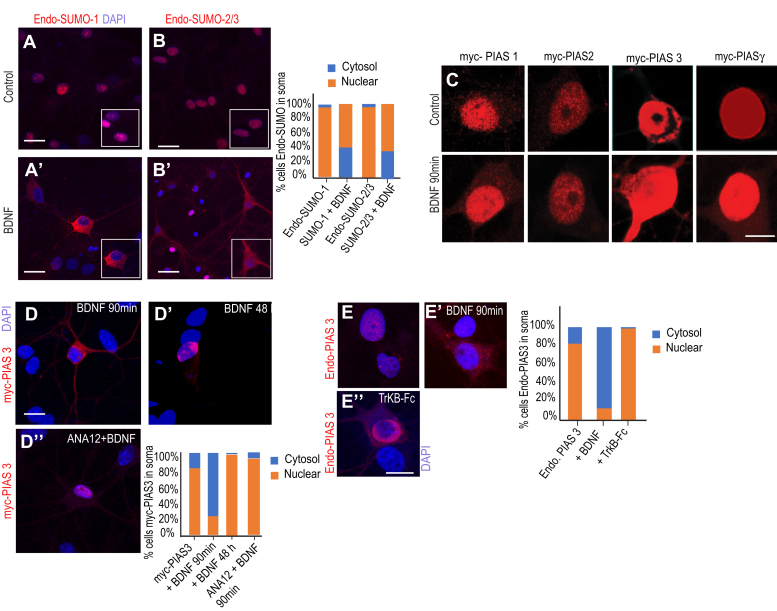
**BDNF alters subcellular localization of SUMO pathway proteins.***A–B*, endogenous SUMO-1 and SUMO-2/3 staining in control neurons. *A′-B′*, endogenous SUMO-1 and SUMO-2/3 staining in BDNF-treated neurons. Quantification of hippocampal neurons exhibiting dendritic/nuclear enrichment of SUMO-1 or SUMO-2/3. *C*, neurons transfected with myc-PIAS-1, myc-PIAS-2α, myc-PIAS-3, or myc-PIAS-γ in control and BDNF-treated neurons. *D-D′*, neurons transfected with myc-PIAS-3 and treated with BDNF (90 min or 48 h). *D’’*, neurons transfected with myc-PIAS-3 and treated with ANA-12 prior to BDNF application. Quantification of nuclear/dendritic enrichment of myc-PIAS-3. *E-E′*, endogenous PIAS-3 showing nuclear localization in control neurons and dendritic enrichment after BDNF application. *E’’*, endogenous PIAS-3 showing nuclear enrichment after TrkB-Fc coapplication with BDNF. Quantification of nuclear/dendritic enrichment of endogenous PIAS-3 after acute BDNF application or TrkB-Fc co-application. Three independent experiments N = 30, Scale bar 5 μm. BDNF, brain-derived neurotrophic factor; PIAS, protein inhibitor of activated STAT; SUMO, small ubiquitin-like modifier; TrkB, tropomyosin-related kinase B.

To test whether BDNF acted as upstream signal to regulate the subcellular localization of SUMO proteins in neurons, we treated primary hippocampal neuronal cultures at 15 days *in vitro* (DIV 15) with BDNF (10 ng/ml, 90 min) followed by immunostaining of endogenous SUMO-1 or SUMO-2/3 (Fig. 1,*A* and *B*’ ). In untreated control neurons, endogenous SUMO-1 and SUMO-2/3 showed a strong nuclear enrichment consistent with previous published reports (Fig. 1, *A* and *B*). However, in contrast, the BDNF-treated neurons showed redistribution of SUMO-1 and SUMO-2/3 to somatic and dendritic compartments (Fig. 1,*A*’-*B*’). Quantification for SUMO-1 [Chi-squared test, χ^2^ (1, N = 100) = 70.09 *p* < 0.00001] or SUMO-2/3 [chi-squared test, χ^2^ (1, N = 100) = 76.62 *p* < 0.00001] subcellular localization changes after BDNF treatment (90 min) confirmed a significant enrichment in the soma and dendrites of the hippocampal neurons.

If the subcellular localization changes in SUMO proteins leads to differential substrate SUMO conjugation, we reasoned that PIAS family of E3 ligase might also exhibit similar somatic enrichment after BDNF treatment. In order to assess this, we transfected DIV 7 neurons with myc-PIAS (myc-PIAS-1, -2, -3 or γ), and at DIV 15, we treated the culture with BDNF (10 ng/ml, 90 min) followed by immunostaining for myc. Of the different PIAS family members tested in our assay, only myc-PIAS-3 showed nucleus to soma translocalization upon BDNF application (Figs. 1*C* and S1). To test whether BDNF-induced myc-PIAS-3 somatic enrichment was acting *via* the TrkB receptor signaling, we treated myc-PIAS-3–transfected primary neurons with BDNF for either 90 min or up to 48 h. At 90 min time point, PIAS-3 was enriched in the soma and dendrites; interestingly, at 48 h time point, we observed enrichment of myc-PIAS-3 within the nucleus (Fig. 1,*D*–*D*’). In order to test if TrkB receptor signaling was necessary to see this relocalization, we treated the primary neurons with the pharmacological TrkB antagonist (ANA-12, 400 nM) 5 min prior to BDNF application. We imaged the cells at 90 min after ANA-12 and BDNF treatment (Fig. 1*D*’’) and found nuclear enrichment of myc-PIAS-3. Quantification confirmed that somatic localization of myc-PIAS-3 is indeed reversible and can be successfully blocked using a pharmacological inhibitor of TrkB (chi-squared test myc-PIAS3 *versus* myc-PIAS3 BDNF treatment, χ^2^ (1, N = 100) = 73.08 *p* < 0.00001). We also assessed if endogenous PIAS-3 somatic underwent relocalization at 90 min post BDNF application (Fig. 1,*E*–*E*’) in order to eliminate any myc-PIAS-3 subcellular localization change after BDNF treatment as an overexpression artefact. Endogenous PIAS-3 was enriched within the nucleus in control untreated neurons. We found that scavenging BDNF using chimeric TrkB-Fc (1 μg/ml) prevented relocalization of endogenous PIAS-3 to the nucleus (Fig. 1*E*’’). However, upon 90 min BDNF application, endogenous PIAS-3 relocalized to soma and dendrites (one-way ANOVA, Dunnett’s test F_(2,9)_ = 303, *p* < 0.0001). Together, our results concur with conclusion that BDNF is a novel and specific regulator of SUMO proteins subcellular localization in neurons.

### SUMO-deficient gephyrin mutants are insensitive to acute BDNF treatment

**Figure 2:**
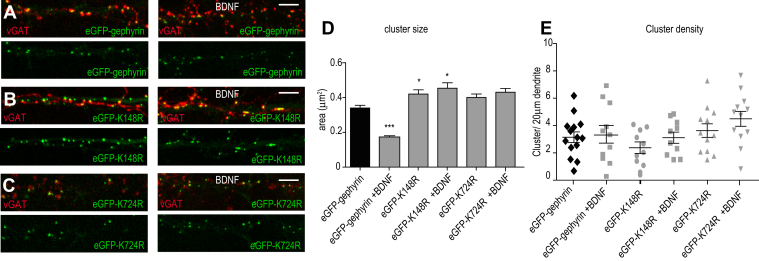
**Gephyrin SUMO-defective mutants do not respond to acute BDNF.***A–C*, morphology of dendritic segments transfected with eGFP-gephyrin or its SUMO-defective mutants K148R or K724R under control conditions and after acute BDNF application. *D*, quantification of eGFP-gephyrin or its mutant cluster size under control conditions or after acute BDNF application. *E*, quantification of eGFP-gephyrin or its mutant cluster density under control conditions or after acute BDNF application. The data were quantified from four independent experiments and 15 neurons/condition. Two-way ANOVA, Bonferroni post hoc comparison. Error bars st.dev. Scale bar 10 μm. ∗*p* < 0.05 and ∗∗∗*p* < 0.0001. BDNF, brain-derived neurotrophic factor; SUMO, small ubiquitin-like modifier.

Acute BDNF treatment through its high-affinity receptor TrkB reduces cell surface expression of α2 GABA_A_R and inhibitory postsynaptic scaffolding protein gephyrin clustering (20). In addition, it has been shown that gephyrin is a substrate for SUMO-1 and SUMO-2/3 conjugation by PIAS-3 (13). Hence, we wondered whether BDNF-induced gephyrin scaffold loss (submembrane lattice or puncta) was facilitated by gephyrin SUMOylation. We transfected primary hippocampal neurons with gephyrin expression constructs that contained either SUMO-1 conjugation–defective mutation (K148R) or SUMO-2 conjugation–defective mutation (K724R) at 7 DIV and treated them with BDNF at 15 DIV after the peak synaptogenesis (Fig. 2, *A*–*C*). We assessed the cells after 90 min for morphological changes in gephyrin cluster size and density. We successfully replicated previous finding (20) reporting a significant reduction in gephyrin cluster size after BDNF (10 ng/ml, 90 min) treatment (0.31 μm^2^ ± 0.02 *versus* 0.18 μm^2^ ± 0.01) (Fig. 2*D*). Under basal conditions, neurons expressing the eGFP-K148R mutant showed larger gephyrin clusters compared to eGFP-gephyrin control (0.42 μm^2^ ± 0.024 *versus* 0.31 μm^2^ ± 0.02). On the other hand, eGFP-K724R gephyrin mutant expressing neurons exhibited cluster size similar (no statistical significance) to eGFP-gephyrin control (0.40 μm^2^ ± 0.02 *versus* 0.31 μm^2^ ± 0.02). Neither eGFP-K148R nor eGFP-K724R mutants showed change in cluster size after 90 min BDNF application (Fig. 2*D*; 0.42 μm^2^ ± 0.024 *versus* 0.41 μm^2^ ± 0.02 and 0.40 μm^2^ ± 0.02 *versus* 0.35 μm^2^ ± 0.02; two-way ANOVA, Bonferroni post hoc test, *p* < 0.0001). Quantification for gephyrin cluster density showed no changes between eGFP-gephyrin, eGFP-K148R, and eGFP-K724R after BDNF application (Fig. 2*E*; two-way ANOVA F_(5, 53)_ = 3.34; Bonferroni post hoc test, *p* = 0.87). Our data show that SUMOylation is an important determinant for increasing gephyrin cluster size to scaffold GABA_A_ receptors at postsynaptic sites within dendrites under BDNF influence.

### BDNF and not NT3 or NT4 influences gephyrin clustering

**Figure 3:**
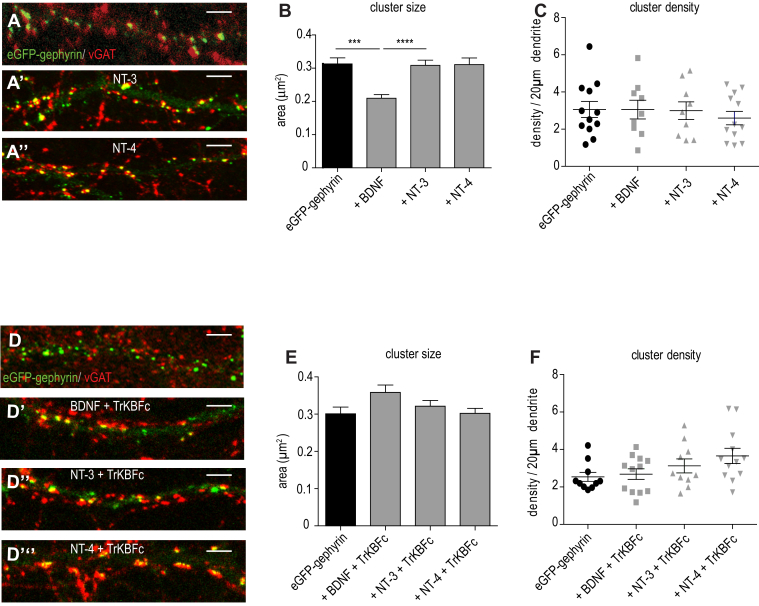
**NT-3 and NT-4 do not impact gephyrin clustering.***A–A’’*, eGFP-gephyrin transfected neurons treated with NT-3 or NT-4 (90 min). *B*, quantification shows BDNF-specific effect on eGFP-gephyrin cluster size reduction. *C*, quantification of eGFP-gephyrin cluster density after BDNF, NT-3, or NT-4 treatments. *D–D’’’*, morphology of denritic segments transfected with eGFP-gephyrin and staining for vGAT presynaptic terminals. *E*, quantification of eGFP-gephyrin cluster size after treating neurons with TrkB-Fc and BDNF, NT-3, or NT-4. *F*, quantification of eGFP-gephyrin cluster density after treating neurons with TrkB-Fc and BDNF, NT-3, or NT-4. The data were quantified from four independent experiments and 15 neurons/condition. Two-way ANOVA, Bonferroni post hoc comparison. Error bars st.dev. Scale bar 10 μm. ∗∗∗*p* < 0.0001 and ∗∗∗∗*p* < 0.00001. BDNF, brain-derived neurotrophic factor; TrkB, tropomyosin-related kinase B.

It is established that both BDNF and NT4 can activate TrkB signaling. Hence, we compared BDNF with NT3 which preferentially activates TrkC and BDNF with NT4 that activates TrkB (23). The activation of signaling cascade downstream of TrkB upon activation by BDNF or NT4 is distinct (24). To understand signaling crosstalk between neurotrophic factors (BDNF, NT3, and NT4) for gephyrin clustering changes, we treated the primary neurons transfected with eGFP-gephyrin with either BDNF, NT-3, or NT-4 (10 ng/ml, 90 min). Quantification for cluster size confirmed that the reduction of eGFP-gephyrin cluster size was specific to BDNF treatment as there were no changes after NT-3 and NT-4 application (Fig. 3*B*) (0.31 μm^2^ ± 0.02 v*ersus* 0.31 μm^2^ ± 0.016 or 0.32 μm^2^ ± 0.02; one-way ANOVA, F_(3,640)_ = 5.19, *p* = 0.0015). On the other hand, quantification for cluster density revealed no changes upon BDNF, NT-3, or NT-4 treatment (Fig. 3*C*; one-way ANOVA, F_(3,38)_ = 0.27, *p* = 0.84). In support of our data showing BDNF signaling specificity, when we scavenged BDNF using TrkB-Fc chimera (90 min), we could prevent gephyrin cluster size reduction (Fig. 3, *D*–*D*’’’ and *E*; two-way ANOVA, F_(3,792)_ = 2.4, *p* = 0.065). The cluster density remained unaffected after TrkB-Fc application (Fig. 3*F*; two-way ANOVA, F_(3,40)_ = 2.33; *p* = 0.09). Consistent to our earlier observations, TrkB-Fc application did not impact eGFP-gephyrin cluster size and density in neurons treated with either NT-3 or NT-4 (Fig. 3, *D*–*F*). The results confirm that BDNF signaling specifically reduces gephyrin cluster size through SUMOylation at K148 and K724 sites respectively.

### ERK1/2 kinase pathway influences PIAS3 effect on gephyrin clustering

**Figure 4:**
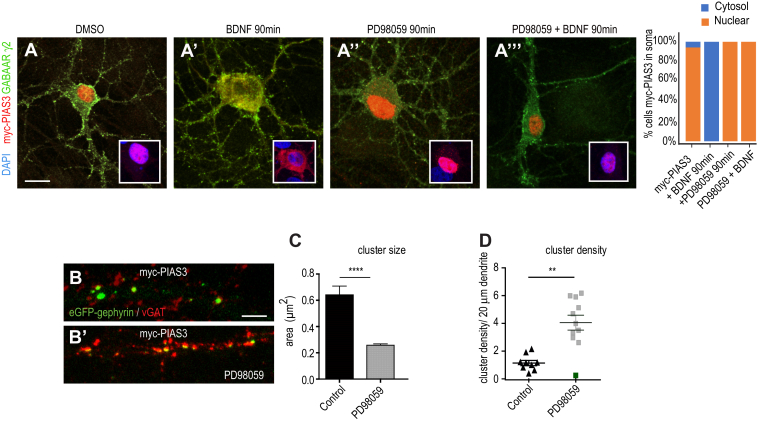
**ERK1/2 regulates PIAS3 subcellular localization.***A–A’’’*, morphology of neuron co-expressing eGFP-gephyrin and myc-PIAS-3 and stained for γ2 GABA_A_Rs. Inset box shows zoom for nuclear enrichment of PIAS-3. The transfected neurons were treated with BDNF, PD98059, or PD98059 together with BDNF (90 min). Quantification shows soma and dendrite enrichment of PIAS-3 is blocked upon pharmacological block of ERK1/2 pathway. The representative data are from four independent experiments. One-way ANOVA and Dunnett’s Test. Error bars SEM. Scale bar 5 μm. *B-B’’’*, morphology of dendritic segment co-expressing eGFP-gephyrin and myc-PIAS-3 treated with ERK1/2 inhibitor PD98059. *C*, quantification of eGFP-gephyrin cluster size in neurons co-expressing myc-PIAS-3 after treatment with PD98059 is reduced. *D*, quantification of eGFP-gephyrin cluster density in neurons co-expressing myc-PIAS-3 after treatment with PD98059 is increased. The representative data are from four independent experiments. Two-tailed Student *t* test. Error bars st.dev. Scale bar 10 μm. ∗∗*p* < 0.01 and ∗∗∗*p* < 0.00001. BDNF, brain-derived neurotrophic factor; PIAS, protein inhibitor of activated STAT.

BDNF signaling activates ERK1/2 downstream of TrkB (25). Gephyrin is an established ERK1/2 substrate and ERK has been reported to phosphorylate gephyrin at S268 residue. The phosphorylation at gephyrin S268 residue has a negative impact on GABAergic transmission (26). Hence, we assessed whether PIAS-3 nuclear to dendrite translocation after BDNF application was mediated by ERK1/2 kinase pathway. Hence, we transfected myc-PIAS3 into primary neurons (DIV7+7) and treated the neurons with BDNF, ERK1/2 inhibitor PD98059 or PD98059 and BDNF. We observed that relocation of myc-PIAS-3 to soma and dendrites after BDNF application was blocked upon pharmacological inhibition of ERK1/2 pathway (Fig. 4, *A*–*A*’’’ ). Quantification confirmed the morphological observation (one-way ANOVA, Dunnett’s test, F_(3,8)_ = 1273, *p* < 0.0001). Our data identified ERK1/2 pathway downstream of BDNF for PIAS-3 subcellular localization change in neurons.

We next examined if ERK1/2 pathway would impact PIAS-3–induced changes in gephyrin clustering. To test this, we treated neurons cotransfected with eGFP-gephyrin and myc-PIAS-3 with pharmacological inhibitor of ERK1/2, PD98059 (25 μM). In comparison to myc-PIAS-3 cotransfected control neurons, PD98059 treatment significantly reduced the size of eGFP-gephyrin clusters (Fig. 4, *B*–*B*’ and *C*; 0.26 μm^2^ ± 0.01 *versus* 0.64 μm^2^ ± 0.068; Kolmogorov–Smirnov test, *p* < 0.0001). In addition, blocking ERK1/2 pathway also increased the density of eGFP-gephyrin clusters in myc-PIAS3 co-expressing cells (Fig. 4*D*; 4.05 ± 0.5 *versus* 1.14 ± 0.19 clusters/20 μm; two-tailed Student *t* test *p* < 0.0001). Our data reveal that the ERK1/2 pathway not only facilitates nucleus to dendrite translocation of PIAS-3 but also regulates the ability of PIAS-3 to influence gephyrin’s cluster size and density.

### PIAS-3 harbors two gephyrin interaction sites

**Figure 5:**
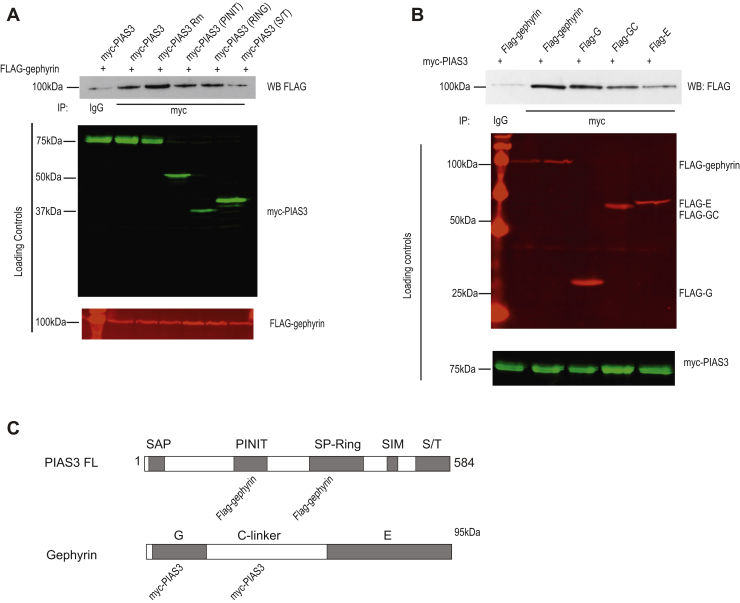
**PIAS-3 and gephyrin interactions occur at two sites.***A*, HEK293 cells cotransfected with Flag-gephyrin and myc-PIAS-3 or myc-PIAS-3 mutations. Immunoprecipitation for myc, followed by Western blot for flag show gephyrin interaction at PINIT and SP-Ring domains within PIAS-3. Protein loading controls are shown below. *B*, HEK293 cells cotransfected with myc-PIAS-3 and Flag-gephyrin or Flag-gephyrin domains. IP for myc, followed by WB for flag show PIAS-3 interaction at G and E domains within gephyrin. Protein loading controls are shown below. IP, immunoprecipitation; PIAS, protein inhibitor of activated STAT; WB, Western blot.

In order to understand the biochemical basis for PIAS-3–mediated gephyrin clustering changes, we assessed for PIAS-3 ability to directly interact with gephyrin. We have previously shown that of the various PIAS family members, only PIAS-3 and PIAS-2α interact with gephyrin (13). Importantly, PIAS-3 interaction with gephyrin is determined by the phosphorylation status of gephyrin at S268 and S270 sites, respectively (13). Here, we assessed for binding domain(s) within PIAS-3 for gephyrin interaction. For this, we cotransfected the HEK293 cells with FLAG-gephyrin and myc-PIAS-3, myc-PIAS-3 RING domain catalytic inactive mutant (Rm), myc-PIAS3 PINIT domain, myc-PIAS3 RING domain, or myc-PIAS3 S/T domain. Immunoprecipitation (IP) for myc-PIAS-3, followed by Western blotting against FLAG-gephyrin confirmed gephyrin and PIAS-3 interaction (Fig. 5*A*; lane 2). In addition, we observed that PIAS-3 domains namely, PINIT domain (1-273) and RING domain (274-392) could interact with gephyrin (Fig. 5*A*; lanes 4–5). These results suggest that gephyrin interaction with PIAS-3 can occur *via* more than one interaction site.

**Figure 6:**
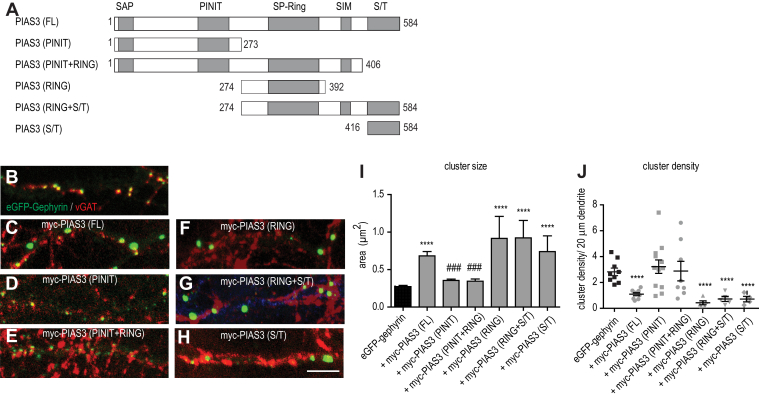
**PIAS3 RING and S/T domains influence gephyrin clustering.***A*, schematic representation of various myc-PIAS-3 deletion mutants used in the study. *B*–*H*, morphology of dendritic segment co-expressing eGFP-gephyrin and various myc-PIAS-3 deletion mutants apposed to vGAT positive terminals. *I*, quantification of eGFP-gephyrin cluster size in neurons co-expressing various myc-PIAS-3 deletion mutations. *J*, quantification of eGFP-gephyrin cluster density in neurons co-expressing various myc-PIAS-3 deletion mutations. The data were quantified from four independent experiments and 15 neurons/condition. ∗∗∗ difference compared to eGFP-gephyrin; ### difference compared to myc-PIAS-3. One-way ANOVA, Error bars st. dev. Scale bar 10 μm. ∗∗∗∗*p* < 0.00001, #### different from other mutants. PIAS, protein inhibitor of activated STAT.

To determine the PIAS-3 interaction sites on gephyrin, we cotransfected HEK293 cells with myc-PIAS-3 and FLAG-gephyrin, FLAG-G, FLAG-GC, or E domain truncation mutant of gephyrin. IP for myc-PIAS-3, followed by Western blotting for FLAG-gephyrin confirmed the previously reported interaction between full-length PIAS-3 and gephyrin (Fig. 5*B*; lane 2). In addition to binding to full-length gephyrin, PIAS-3 interaction was seen with FLAG-G, FLAG-GC, and FLAG-E domain truncation mutations of gephyrin (Fig. 6*B*; lanes 3–5). Our biochemical data are consistent with the earlier observation that PIAS-3 SUMOylates gephyrin at K148 and K724 sites located on the G- and E-domain, respectively (13).

### PIAS-3 domains regulate gephyrin clustering independent of each other

Given the biochemical interaction of gephyrin with more than one site on PIAS-3, we wanted to assess the influence of the five conserved domains within PIAS3, namely SAP, PINIT, SP-Ring, SIM, and Ser/Thr (S/T) rich, on eGFP-gephyrin clustering (Fig. 6*A*). It has been previously reported that the RING domain is important for the SUMO E3 ligase function of PIAS-3 (27). In addition to the RING domain, N-terminus PINIT domain also contributes to substrate SUMOylation (28, 29). The SAP domain facilitates PIAS-3 DNA binding (30), and function of S/T domain remains unclear.

We used myc-PIAS-3 deletion mutants that have been previously described (31) to examine the function of each of these five domains in neurons (Fig. 6*A*). We cotransfected primary neuron with full-length myc-PIAS-3 (1-584), myc-PIAS-3 SAP and PINIT domain (1-273), myc-PIAS-3 SAP, PINIT, SP-Ring and SIM domains (1-406), myc-PIAS-3 SP-Ring domain (274-392), myc-PIAS3 SP-Ring, SIM and S/T domains (274-584), or myc-PIAS-3 S/T domain (416-584), along with eGFP-gephyrin (Fig. 6, *B*–*H*). We assessed for changes in eGFP-gephyrin cluster size compared to the full-length myc-PIAS-3 and eGFP-gephyrin controls. Consistent with an earlier report, we found that myc-PIAS-3 co-expression significantly increased eGFP-gephyrin cluster size compared to neurons transfected with eGFP-gephyrin alone (Fig. 6*C*; 0.68 μm^2^ ± 0.06 *versus* 0.27 μm^2^ ± 0.02). Neurons co-expressing myc-PIAS-3 N terminus (1–273AA) did not increase the size of eGFP-gephyrin clusters (0.36 μm^2^ ± 0.02 *versus* 0.27 μm^2^ ± 0.02). Similarly, neurons co-expressing myc-PIAS-3 (1-406) had no change in eGFP-gephyrin cluster size (0.27 μm^2^ ± 0.02 *versus* 0.34 μm^2^ ± 0.03 and 0.68 μm^2^ ± 0.06). However, neurons expressing the PIAS-3 RING-domain fragment (274-392) showed a significantly increased eGFP-gephyrin cluster size similar to full-length PIAS-3 (0.68 μm^2^ ± 0.06 and 0.9 μm^2^ ± 0.3 *versus* 0.27 μm^2^ ± 0.02). The co-expression of myc-PIAS-3 (274-584) or myc-PIAS-3 S/T domain (416-584) also significantly increased eGFP-gephyrin cluster size (0.9 μm^2^ ± 0.2 *versus* 0.27 μm^2^ ± 0.02 and 0.74 μm^2^ ± 0.2 *versus* 0.27 μm^2^ ± 0.02; one-way ANOVA, Bonferroni post hoc pair wise comparison, F_*(6, 511)*_ = 20.40; *p <* 0.0001).

Next, we investigated if there were changes in eGFP-gephyrin cluster density in neurons co-expressing different myc-PIAS-3 deletion mutants. The co-expression of full-length myc-PIAS-3 significantly reduced eGFP-gephyrin cluster density (Fig. 6*J*; 1.09 ± 0.12 *versus* 2.8 ± 0.29 clusters/20 μm). The co-expression of either myc-PIAS-3 (1-273) (3.22 ± 0.52 *versus* 2.8 ± 0.29 clusters/20 μm) or myc-PIAS-3 (1-406) containing the PINIT and SP-RING domains showed no change in eGFP-gephyrin cluster density (2.74 ± 0.46 *versus* 2.89 ± 0.76 clusters/20 μm). On the other hand, neurons co-expressing the myc-PIAS-3 (273-392) SP-RING domain showed a significant reduction of eGFP-gephyrin cluster density (0.43 ± 0.15 *versus* 2.8 ± 0.29 clusters/20 μm). We also observed a significant reduction in eGFP-gephyrin cluster density in neurons co-expressing two different C-terminus fragments, myc-PIAS-3 (274-584) or myc-PIAS-3 (416-584) (one-way ANOVA, Bonferroni post hoc pair wise comparison, F_*(6,26)*_ = 20.89; *p <* 0.0001). Our analysis of PIAS-3 domains identifies a role for RING (274-392) domain in increasing the gephyrin cluster size the and S/T domain (416-584) in decreasing the gephyrin cluster density. However, PINIT domain can partially block the RING domain function.

### BDNF signaling regulates RING and S/T domain functions

**Figure 7:**
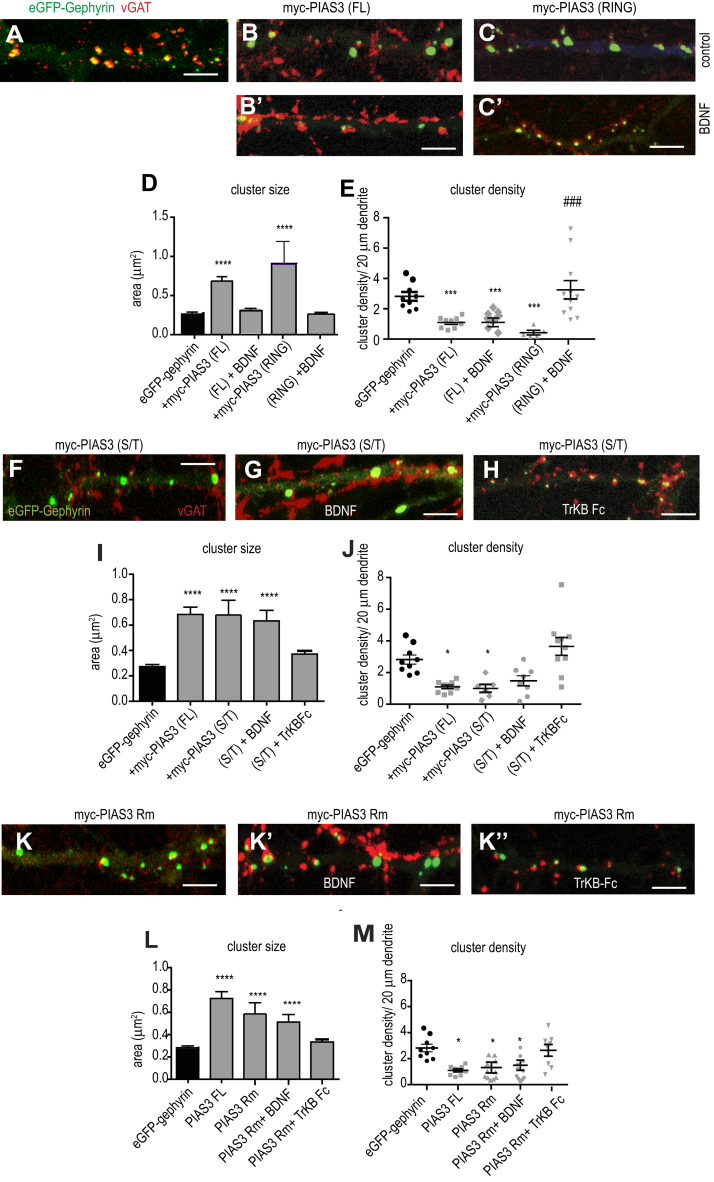
**BDNF signaling differentially regulates SP-RING and Ser/Thr rich domain functions.***A–C′*, morphology of dendritic segments showing eGFP-gephyrin and myc-PIAS-3 or myc-PIAS-3 RING domain in control and BDNF-treated neurons. *D*, quantification of eGFP-gephyrin cluster size in control and BDNF-treated neurons co-expressing myc-PIAS-3 or myc-SP-RING domain. *E*, quantification of eGFP-gephyrin cluster density in control and BDNF-treated neurons co-expressing myc-PIAS-3 or myc-SP-RING domain. *F*–*H*, morphology of dendritic segment expressing eGFP-gephyrin and myc-PIAS-3 S/T domain and treated with BDNF or TrkB-Fc. *I*, quantification of eGFP-gephyrin cluster size in eGFP-gephyrin control and myc-PIAS-3 S/T co-expressing neurons after BDNF or TrkB-Fc application. *J*, quantification of eGFP-gephyrin cluster density in eGFP-gephyrin alone or myc-PIAS-3 S/T co-expressing neurons after BDNF or TrkB-Fc application. *K-K’’*, morphology of dendritic segment expressing eGFP-gephyrin and myc-PIAS-3Rm mutant and treated with BDNF or TrkB-Fc. *L*, quantification of eGFP-gephyrin cluster size in eGFP-gephyrin control and myc-PIAS-3Rm co-expressing neurons after BDNF or TrkB-Fc application. *M*, quantification of eGFP-gephyrin cluster density in eGFP-gephyrin alone or myc-PIAS-3Rm co-expressing neurons after BDNF or TrkB-Fc application. The data were quantified from four independent experiments and 15 neurons/condition. Two-way ANOVA, Bonferroni post hoc comparison. Error bars st.dev. Scale bar 10 μm. ∗*p* < 0.05, ∗∗∗*p* < 0.0001, and ∗∗∗∗*p* < 0.00001; ### different from other mutants. BDNF, brain-derived neurotrophic factor; PIAS, protein inhibitor of activated STAT; TrkB, tropomyosin-related kinase B.

To understand whether BDNF regulates PIAS-3 *via* both the RING and S/T domains, we cotransfected neurons with eGFP-gephyrin and full-length (FL) myc-PIAS-3 (1-584) or myc-PIAS-3 S/T (274-392). We then treated the cotransfected neurons with BDNF for 90 min and morphologically analyzed for alteration in eGFP-gephyrin cluster size and density at DIV 15 (Fig. 7). Quantification confirmed that myc-PIAS-3 FL and myc-PIAS-3 RING co-expressing neurons increased the eGFP-gephyrin cluster size in comparison to eGFP-gephyrin control neurons (Fig. 7, *A*–*D*; 0.26 μm^2^ ± 0.02 *versus* 0.9 μm^2^ ± 0.3, one-way ANOVA Bonferroni pair wise comparison, F_(4,430)_ = 15.3, *p* < 0.0001). The increase of eGFP-gephyrin cluster size upon myc-PIAS-3 FL or myc-PIAS-3 RING co-expression was however not evident after BDNF application (Fig. 7*D*), suggesting that BDNF directly influences PIAS-3 function to influence gephyrin cluster alteration. Quantification for eGFP-gephyrin cluster density showed significant reduction in upon myc-PIAS-3 FL or myc-PIAS-3 RING co-expression (Fig. 7*E*). The application of BDNF 90 min was not sufficient to normalize the eGFP-gephyrin cluster density in neurons co-expressing myc-PIAS-3 FL (Fig. 7*E*). However, eGFP-gephyrin cluster density was normalized in neurons co-expressing PIAS-3 RING after BDNF treatment (3.25 ± 0.6 *versus* 0.73 ± 0.19 clusters/20 μm) (one-way ANOVA, Bonferroni pair wise comparison, F_*(4, 32)*_ = 18.89; *p <* 0.001). Together, our results identify that BDNF impacts RING domain function for increasing gephyrin cluster size and reducing cluster density number.

We next quantified the effect of myc-PIAS-3 S/T expression on eGFP-gephyrin clustering. We treated the neurons with BDNF and found no change to the size or density of gephyrin clusters. Therefore, we tested TrkB-Fc in these transfected cells and analyzed for morphological changes in gephyrin cluster size and density at DIV 15 (Fig. 7, *F*–*H*). Quantification confirmed that morphological changes induced by PIAS-3 S/T domain on eGFP-gephyrin cluster size was not impacted by BDNF treatment (Fig. 7*I*; 0.6 μm^2^ ± 0.08 *versus* 0.79 μm^2^ ± 0.15, one-way ANOVA, Bonferroni post hoc pair wise comparison, F_(4,520)_ = 14.87, *p* < 0.0001). Similarly, eGFP-gephyrin cluster density in neurons co-expressing PIAS-3 S/T domain was not impacted after BDNF treatment (Fig. 7*J*; 1.5 ± 0.3 *versus* 1 ± 0.3 clusters/20 μm, one-way ANOVA, Bonferroni post hoc pair wise comparison, F_(4,26)_ = 17.45, *p* < 0.05). However, upon analysis of neurons that were treated with TrkB-Fc, we found that eGFP-gephyrin cluster size returned to base line levels (Fig. 7*I*; 0.41 μm^2^ ± 0.003 *versus* 0.79 μm^2^ ± 0.15). Similarly, quantification for gephyrin cluster density in neurons co-expressing PIAS-3 S/T returned to base line level after TrkB-Fc treatment (Fig. 7*J*; 3.8 ± 0.5 *versus* 1 ± 0.3 clusters/20 μm). These data show that S/T domain function is regulated in a mechanism opposite to RING domain function, which requires active BDNF signaling.

To understand this discrepancy in BDNF-mediated PIAS-3 regulation better, we used myc-PIAS3Rm wherein the RING domain in FL PIAS-3 has been rendered catalytically inactive by mutations (C299S/H301A) (31). Given that the PIAS-3Rm is defective for SUMO conjugation, we did not expect a phenotype change in eGFP-gephyrin clustering. However, cluster size of eGFP-gephyrin was increased in neurons transfected with myc-PIAS-3Rm and cluster density was reduced as seen with wild type myc-PIAS-3. To better understand how myc-PIAS-3Rm altered eGFP-gephyrin clustering, we treated neurons co-transfected with myc-PIAS-3Rm with BDNF (90 min). BDNF application did not change the morphology of eGFP-gephyrin in neurons co-expressing myc-PIAS-3Rm (Fig. 7, *K*-*K*’). eGFP-gephyrin cluster size of treated neurons expressing myc-PIAS-3Rm with TrkB-Fc were similar control eGFP-gephyrin–only transfected neurons (Fig. 7, *K*’’ and L, one-way ANOVA, Bonferroni post hoc pair wise comparison, F_(4, 230)_ = 20.21, *p* < 0.0001). Similarly, treatment with TrkB-Fc normalized gephyrin clusters in neurons cotransfected with myc-PIAS-3 Rm (Fig. 7*M*, one-way ANOVA, Bonferroni post hoc pair wise comparison, F_(4, 20)_ = 13.67, *p* < 0.05).

Together, our data show that BDNF *via* TrkB signaling regulates PIAS-3 RING domain function, while scavenging BDNF impacts S/T domain function.

### PIAS-3 impairs GABAergic transmission

**Figure 8:**
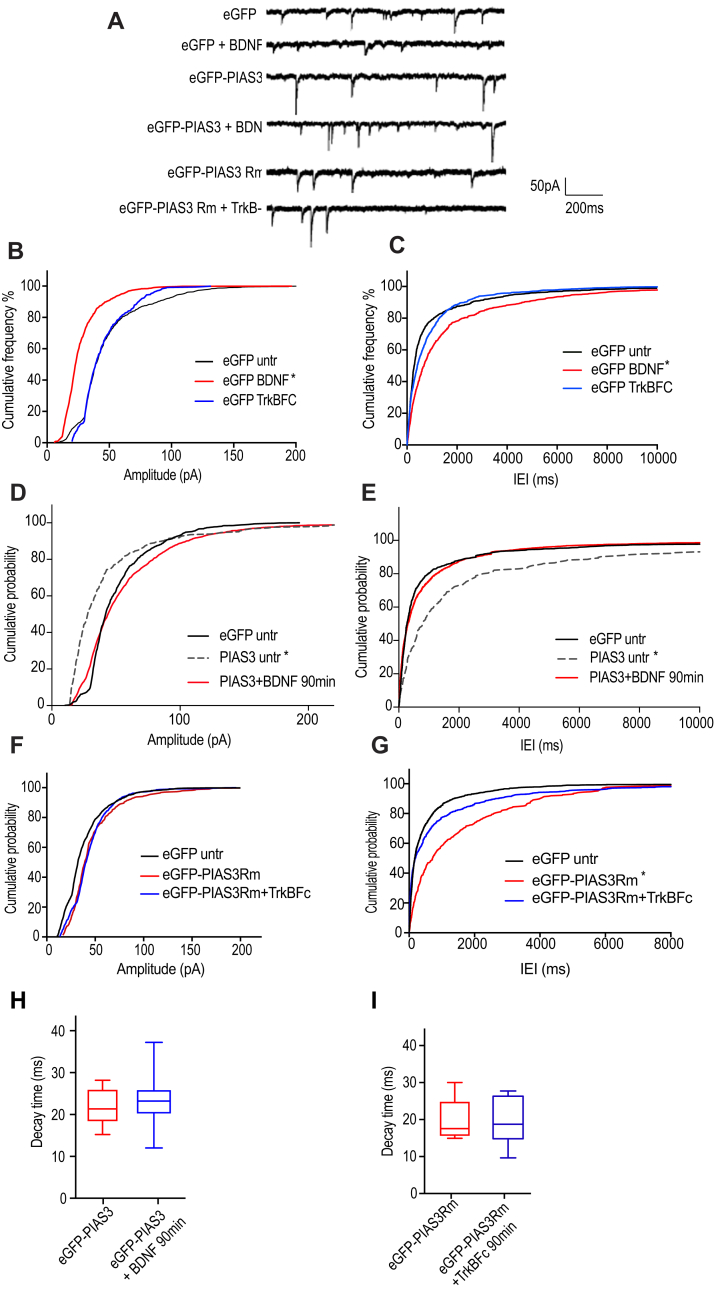
**Effects of eGFP-PIAS-3 on GABAergic mIPSCs in cultured hippocampal neurons.***A*, representative current traces show pharmacologically isolated GABAergic mIPSCs recorded at 11 + 3 DIV. *B*, cumulative probability distribution of the average amplitude of mIPSCs from eGFP-transfected neurons either treated with BDNF or TrkB-Fc (90 min; n = 15). *C*, cumulative probability distribution of the average interevent intervals of mIPSCs from eGFP-transfected neurons either treated with BDNF or TrkB-Fc (90 min). *D*, cumulative probability distribution of the average amplitude of mIPSCs from eGFP or eGFP-PIAS-3–transfected neurons treated with BDNF (90 min; n = 15). *E*, cumulative probability distribution of the average interevent intervals of mIPSCs from eGFP or eGFP-PIAS-3–transfected neurons treated with BDNF (90 min). *F*, cumulative probability distribution of the average amplitude of mIPSCs in eGFP or eGFP-PIAS-3Rm mutant–transfected neurons treated with TrkB-Fc (90 min; n = 11). *G*, cumulative probability distribution of the average interevent intervals of mIPSCs in eGFP-PIAS-3Rm–transfected neurons treated with TrkB-Fc (90 min). *H*, average current decay time in neurons transfected with either eGFP-PIAS-3 or eGFP-PIAS-3 treated with BDNF. *I*, average current decay time in neurons transfected with either eGFP-PIAS-3Rm or eGFP-PIAS-3Rm treated with TrkB-Fc. Data were collected from three independent experiments. Error bars st.dev. ∗*p* < 0.05. BDNF, brain-derived neurotrophic factor; mIPSCs, miniature inhibitory postsynaptic currents; PIAS, protein inhibitor of activated STAT; TrkB, tropomyosin-related kinase B.

The functional relevance of BDNF, TrkB-Fc, PIAS-3, and PIAS-3Rm on GABAergic neurotransmission was determined by whole-cell patch clamp recordings. We pharmacologically isolated GABAergic miniature inhibitory postsynaptic currents (mIPSCs) in 11 + 4 DIV hippocampal neurons in the presence of sodium channel blocker tetrodotoxin. We first assessed the effect of BDNF or TrkB-Fc on GABAergic neurotransmission (Fig. 8, *A*–*C*). Consistent with the previous publication (20), BDNF treatment (10 ng/ml 90 min) significantly reduced mIPSC amplitude (48.4 ± 0.8pA *versus* 27.9 ± 0.6pA; *p* < 0.05, Kolmogorov–Smirnov test) and increased the interevent intervals (1067.2 ± 91.4 *versus* 1795.9 ± 165.4; *p* < 0.05, Kolmogorov–Smirnov test), suggesting reduced number of GABA_A_Rs at synaptic sites and reduced synapse number. In contrast, scavenging BDNF using TrkB-Fc chimera (1 μg/ml) did not alter mIPSC amplitude or interevent intervals (Fig. 8, *A*–*C*).

As a next step, we overexpressed WT eGFP-PIAS-3 and treated cells with BDNF to evaluate its direct impact on PIAS-3 function and gephyrin clustering. In eGFP-PIAS-3–transfected control neurons, we saw a significant reduction of mIPSC amplitude (66.1 ± 2.4pA *versus* 57.4 ± 0.9pA; *p* < 0.05, Kolmogorov–Smirnov test) and significant increase in mIPSC interevent interval (1984.2 ± 128.5 *versus* 1024.1 ± 54.4; *p* < 0.05, Kolmogorov–Smirnov test) (Fig. 8, *D* and *E*), suggesting reduced GABA_A_Rs at synaptic sites and reduced density of GABAergic synapses. However, BDNF application reversed the eGFP-PIAS-3 effect on GABAergic inhibition and returned mIPSc interevent interval to baseline levels as seen in the mock-transfected control cells. BDNF translocates PIAS-3 from the nucleus to dendrites, and BDNF resets gephyrin cluster size but not cluster density in PIAS-3 overexpressing neurons. In contrast, our functional data suggest that perhaps gephyrin-independent GABA_A_Rs facilitate inhibitory neurotransmission when PIAS-3 SUMOylates gephyrin to prevent macroclustering (oligomerization) at synaptic sites. Similar compensation in GABAergic inhibition has been reported upon ablation of α2 GABA_A_R subunit containing GABA_A_Rs in hippocampal pyramidal neurons (32).

As a next step, we assessed the influence of PIAS-3Rm on GABAergic transmission. We compared differences in mIPSC amplitude between eGFP, eGFP-PIAS-3Rm, or eGFP-PIAS3Rm treated with TrkB-Fc (Fig. 8*F*). Although PIAS-3Rm mutant increases eGFP-gephyrin cluster size morphologically, at a functional level, mIPSC amplitude is not altered (56.9 ± 3.9 *versus* 57.2 ± 1.9). This suggests that gephyrin-independent GABA_A_Rs contribute to the amplitude, while the large gephyrin aggregates observed in PIAS-3Rm-transfected dendrites are perhaps cytosolic protein aggregates due to SUMOylation defect. The eGFP-PIAS-3Rm–expressing cells show shorter (45%) mIPSC interevent intervals (Fig. 5*G*; 6124 ± 473 *versus* 3505 ± 352; *p* < 0.05, Kolmogorov–Smirnov test), suggesting reduced number of GABAergic synapses. The reduced interevent intervals is consistent with the morphological reduction in gephyrin cluster density in myc-PIAS-3Rm expressing neurons (Fig. 7, *K*–*M*). Scavenging BDNF using TrkB-Fc showed interevent intervals similar to eGFP control cells (Fig. 8*G*). The composition of the GABA_A_R subunits can be ascertained by analyzing the rise and decay kinetics of GABAergic mIPSCs. Analyses of rise and decay kinetics of GABAergic mIPSC showed no differences between eGFP-PIAS-3–transfected cells undergoing mock or BDNF treatment (Fig. 8*H*). Our analysis showed no differences in rise and decay times between eGFP-PIAS-3Rm–transfected cells undergoing mock or TrkB-Fc treatment (Fig. 8*I*). Overall, PIAS-3 impairs GABAergic synaptic transmission by reducing GABAergic mIPSC amplitude and synapse density. The negative effect of PIAS-3 on GABAergic transmission is reversed by BDNF signaling. In the PIAS3Rm, RING domain is rendered inactive for SUMO conjugation, whereby not impacting gephyrin clustering. However, we observe functional impact on GABAergic transmission. We concur that perhaps in the absence of the functional RING domain, another domain such as the S/T domain might influence gephyrin clustering abilities to impact GABAergic inhibition.

### ERK1/2 pathway blocks PIAS3Rm and S/T function to restore gephyrin clustering

**Figure 9:**
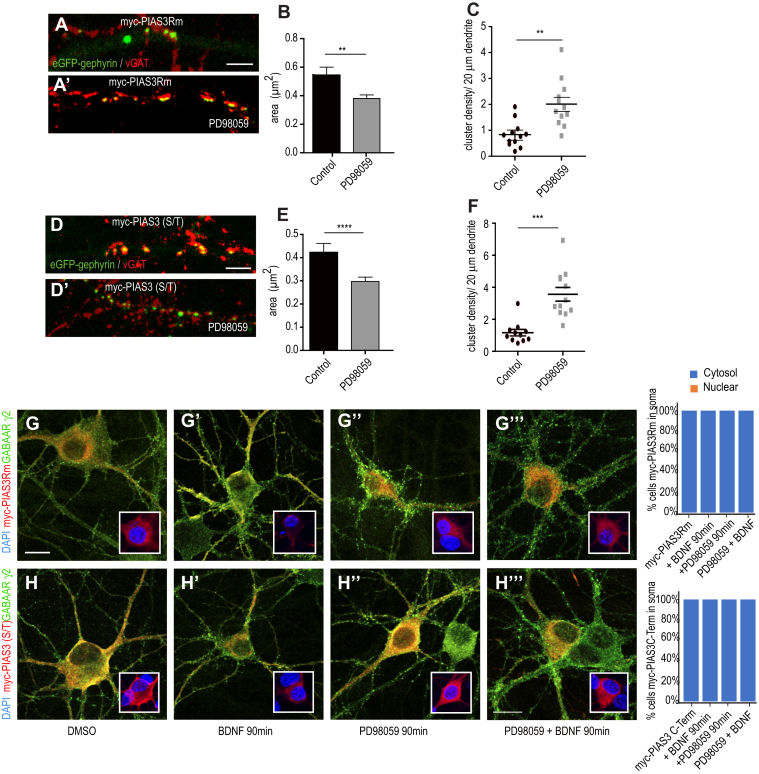
**ERK1/2 pathway regulates PIAS-3 RING domain and S/T domain function.***A–A′*, morphology of dendritic segment co-expressing eGFP-gephyrin and myc-PIAS-3Rm with or without treatment with ERK1/2 inhibitor PD98059 (25 μM). *B*, quantification of eGFP-gephyrin cluster size in neurons co-expressing myc-PIAS-3Rm after treatment with PD98059 is reduced. *C*, quantification of eGFP-gephyrin cluster density in neurons co-expressing myc-PIAS-3Rm after treatment with PD98059 is increased. *D-D′*, morphology of dendritic segment co-expressing eGFP-gephyrin and myc-PIAS-3 S/T with and without treatment with PD98059. *E*, quantification of eGFP-gephyrin cluster size in neurons co-expressing myc-PIAS-3 S/T after treatment with PD98059 is reduced. *F*, quantification of eGFP-gephyrin cluster density in neurons co-expressing myc-PIAS-3 S/T after treatment with PD98059 is increased. The representative data are from four independent experiments. Two-tailed Student *t* test. Error bars st.dev. Scale bar 10 μm. *G-G’’’*, morphology of dendritic segment co-expressing eGFP-gephyrin and myc-PIAS-3Rm and γ2 GABA_A_Rs. Inset box shows zoom for soma/dendrite enrichment of PIAS-3Rm. The transfected neurons were treated with BDNF, PD98059, or PD98059 together with BDNF (90 min). Quantification shows soma and dendrite enrichment of PIAS-3 is unaffected upon pharmacological block of ERK1/2 pathway. *H–H’’’*, morphology of dendritic segment co-expressing eGFP-gephyrin and myc-PIAS-3 S/T domain (416-584) and γ2 GABA_A_Rs. Inset box shows zoom for soma enrichment of PIAS-3. The transfected neurons were treated with BDNF, PD98059, or PD98059 together with BDNF (90 min). Quantification shows soma and dendrite enrichment of PIAS-3 C-terminus domain is not affected upon pharmacological block of ERK1/2 pathway. The representative data are from four independent experiments. One-way ANOVA and Dunnett’s test. Error bars st.dev. Scale bar 5 μm. ∗∗*p* < 0.01, ∗∗∗*p* < 0.0001 and ∗∗∗∗*p* < 0.00001. BDNF, brain-derived neurotrophic factor; mIPSCs, miniature inhibitory postsynaptic currents; PIAS, protein inhibitor of activated STAT.

As it is known that BDNF signaling activates ERK1/2 downstream of TrkB (25), we investigated whether ERK1/2 kinase pathway influenced PIAS-3Rm and S/T domain function in neurons. We treated neurons cotransfected with eGFP-gephyrin and myc-PIAS-3Rm mutant or myc-PIAS-3 S/T (416-584) with pharmacological ERK1/2 inhibitor PD98059 (25 μM). The PD98059 treatment significantly reduced the size of eGFP-gephyrin clusters in neurons expressing myc-PIAS-3Rm (Fig. 9, *A* and *B*; 0.38 μm^2^ ± 0.024 *versus* 0.54 μm^2^ ± 0.56; Kolmogorov–Smirnov test, *p* = 0.009) and increased the cluster density (Fig. 9*C*; 2.01 ± 0.26 *versus* 0.83 ± 0.14 clusters/20 μm; two-tailed Student *t* test *p* < 0.0001). These findings confirmed to us that ERK1/2 pathway regulates PIAS-3Rm function perhaps *via* the S/P domain regulation. To confirm this, we cotransfected eGFP-gephyrin and myc-PIAS-3 S/T domain and treated the cells with PD98059. In cells co-expressing myc-PIAS-3 S/T domain, PD98059 treatment significantly reduced eGFP-gephyrin cluster size (Fig. 9, *D* and *E*; 0.3 μm^2^ ± 0.02 *versus* 0.42 μm^2^ ± 0.04; Kolmogorov–Smirnov test, *p* = 0.02). Similarly, PD98059 treatment increased the density of eGFP-gephyrin clusters in myc-PIAS-3 S/T–expressing cells (Fig. 9*F*; 3.5 μm^2^ ± 0.43 *versus* 1.17 μm^2^ ± 0.2 clusters/20 μm; two-tailed Student *t* test *p* = 0.0006). This confirms that ERK1/2 signaling regulates PIAS-3 S/T domain to influence gephyrin clustering and GABAergic inhibition.

Given that pharmacological blockade of ERK1/2 pathway prevents BDNF-induced PIAS-3 localization to soma and dendrites (Fig. 4*A*), we tested to see if ERK1/2 pathway also influenced PIAS-3Rm and myc-PIAS-3 S/T localization in neurons. For this, we transfected myc-PIAS-3Rm or myc-PIAS-3 S/T domain into primary neurons. We treated the neurons with BDNF, PD98059, or PD98059 and BDNF. Unlike myc-PIAS3 which localizes within the nucleus, the myc-PIAS-3 Rm localized outside the nucleus in soma and dendrites (Fig. 8*B*). After BDNF application, myc-PIAS3 Rm did not relocate to the nucleus (Fig. 9, *G*–*G*’). Similarly, application of PD98059 did not relocate myc-PIAS-3Rm to the nucleus (Fig. 9*G*’’), and co-application of PD98059 and BDNF also did not show nuclear enrichment of myc-PIAS3 Rm (Fig. 9*G*’’’). This suggested to us that myc-PIAS3 Rm is not sensitive to ERK1/2 pathway. Similarly, myc-PIAS-3 S/T domain localizes within soma and dendrites (Fig. 9*H*). Upon BDNF or PD98059 application, we did not see it relocate to the nucleus (Fig. 9,*H*’-H’’). Similarly, co-application of PD98059 and BDNF did not influence the subcellular localization of myc-PIAS-3 S/T (Fig. 9*H*’’’).

### ERK1/2 phosphorylation of gephyrin at S268 impairs PIAS3 influence on clustering

We have reported earlier that gephyrin is a direct substrate for ERK1/2 phosphorylation and that ERK phosphorylation at S268 residue results in reduced gephyrin cluster size, causing a functional reduction in GABAergic inhibition (26). Given our data that ERK pathway directly influences PIAS-3 function, we assessed for crosstalk between gephyrin phosphorylation at S268 and PIAS-3. For this, we transfected primary neurons with eGFP-gephyrin and myc-PIAS-3, myc-PIAS3Rm, myc-PIAS3 RING, or myc-PIAS3 S/T to assess their influence on gephyrin clustering in the presence of PD98059. At a morphological level, the expression of either myc-PIAS-3, myc-PIAS3Rm, myc-PIAS3 RING, or myc-PIAS3 S/T and treatment with PD98059 reduced the intracellular gephyrin aggregates and formed smaller submembrane clusters (Fig. S2, *A*–*H*). Similarly, PD98059 treatment facilitated the formation of numerous eGFP-gephyrin clusters to increase the density significantly (Fig. S2, *C*–*H*). However, specifically in neurons expressing myc-PIAS-3, PD98059 treatment did not increase the density of eGFP-gephyrin clusters (Fig. S2*A*). Overall, we uncover a direct link between gephyrin phosphorylation at S268 residue and PIAS-3–mediated SUMO conjugation on gephyrin.

### Oxygen–glucose deprivation induces downregulation of gephyrin scaffolding and GABAergic inhibition

**Figure 10:**
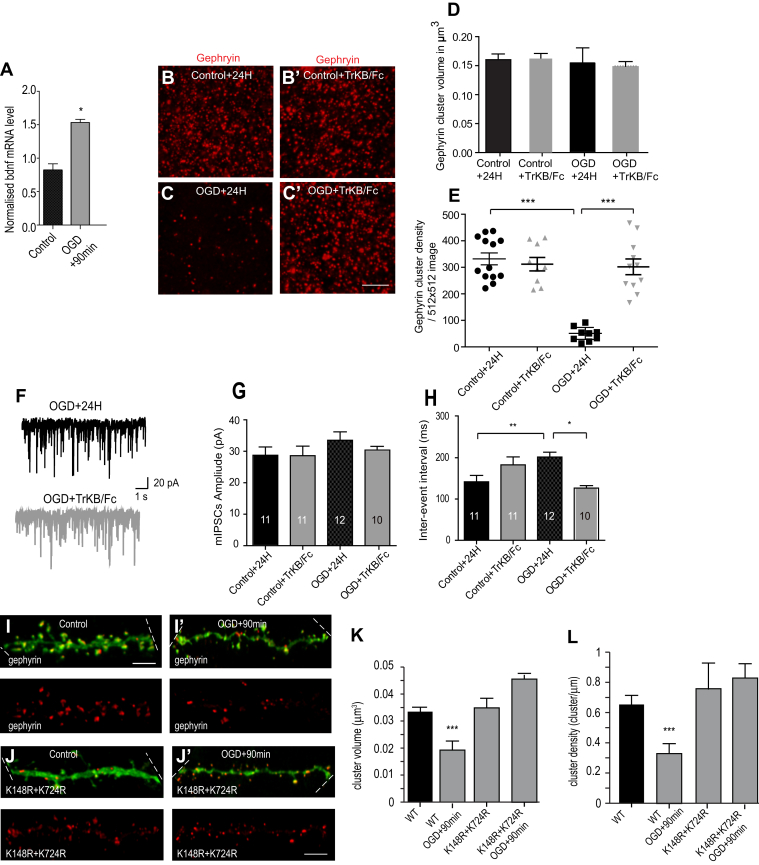
**BDNF promotes gephyrin cluster loss and reduced GABAergic inhibition after OGD.***A*, qPCR of *bdnf* transcript from CA1 area of hippocampus after 90 min after OGD. *B-C′*, morphology of gephyrin clusters in organotypic hippocampus CA1 area in control mock-treated slices, control slices treated with TrkB-Fc, OGD slices after 24 h recovery, OGD slices treated with TrkB-Fc after 24 h recovery (Scale bar 2 μm). *D*, quantification of gephyrin cluster volume in control and OGD slices. *E*, quantification of gephyrin cluster density in control and OGD slices. *F*, representative current traces show pharmacologically isolated GABAergic mIPSCs in OGD slices after 24 h recovery or OGD slices treated with TrkB-Fc in presence of tetrodotoxin. *G*, mIPSC mean amplitude at 24 h post OGD under different conditions tested. *H*, mIPSC interevent interval at 24 h post OGD under different conditions tested. *I-J′*, morphology of pyramidal neurons co-transfected with td-Tomato (*green*) and eGFP-gephyrin (*red*), or eGFP-gephyrin K148R/K724R SUMO-defective mutation in mock-treated or OGD slices after 90 min recovery. Boundaries of the neuronal dendrites within the panel are indicated with *dashed white line*. *K*, quantification of eGFP-gephyrin cluster size in pyramidal neurons co-expressing td-Tomato and eGFP-gephyrin or eGFP-gephyrin K148R/K724R SUMO-defective mutation under control or in OGD slices after 90 min recovery. *L*, quantification of eGFP-gephyrin cluster density in pyramidal neurons co-expressing td-Tomato and eGFP-gephyrin or eGFP-gephyrin K148R/K724R SUMO-defective mutation under control or in OGD slices after 90 min recovery. Data were collected from three independent experiments. Error bars st.dev. Scale bar 5 μm. ∗*p* < 0.05, ∗∗*p* < 0.01, and ∗∗∗*p* < 0.0001. BDNF, brain-derived neurotrophic factor; mIPSCs, miniature inhibitory postsynaptic currents; OGD, oxygen–glucose deprivation; SUMO, small ubiquitin-like modifier; TrkB, tropomyosin-related kinase B.

Independent reports have shown BDNF and global SUMO upregulation under ischemic conditions (16, 33). Rapid internationalization of GABA_A_Rs after ischemia has also been reported (22). We speculated that during ischemia, increased BDNF expression could reduce synaptic abundance of GABA_A_Rs *via* PIAS-3–mediated gephyrin modification at K148 and K724 residues. To test our idea, we used organotypic hippocampal slice culture, as the local neuronal network is well preserved in this *in-vitro* system. We focused on CA1 pyramidal neurons as they have been reported to be more susceptible to ischemia (34). We induced OGD for 4 min and analyzed for BDNF upregulation after 90 min. We performed quantitative real-time PCR (RT-qPCR) analysis to measure change in the *bdnf* transcript at 90 min post OGD. Analysis upon normalization using the house-keeping gene GAPDH showed a significant increase in *bdnf* mRNA levels (Fig. 10*A*; *p* = 0.046). Next, we stained for gephyrin and analyzed for changes in cluster size and density at 24 h post-OGD (Fig. 10, *B* and *C*). Quantification confirmed that at 24 h post-OGD, gephyrin cluster volume is not changed. We also blocked BDNF signaling using TrkB-Fc and assessed for morphological changes in gephyrin clustering (Fig. 10, *D* and *E*). Quantification confirmed that blocking BDNF signaling using TrkB-Fc does not impact gephyrin cluster volume at 24 h post-OGD (Fig. 10*D*; 0.096 μm^3^ ± 0.008 *versus* 0.091 μm^3^ ± 0.006; two-tailed Mann–Whitney *t* test *p* = 0.63). However, at 24 h post-OGD, gephyrin cluster density was significantly reduced. Importantly, TrkB-Fc treatment of OGD slices could prevent the loss of gephyrin clusters (Fig. 10*E*; 47.44 ± 8.78 *versus* 331.9 ± 22.37; two-tailed Mann–Whitney *t* test *p* < 0.0001).

We next examined whether morphological loss of gephyrin cluster density at 24 h post-OGD also resulted in functional loss of GABAergic transmission. For this, we performed whole-cell patch clamp recording GABAergic mIPSC in organotypic slices that were mock treated, treated with TrkB-Fc, underwent OGD, or underwent OGD in the presence of TrkB-01Fc (Fig. 10, *F*–*H*). Consistent with the morphology which showed that gephyrin cluster volume is not changed at 24 h post-OGD, mIPSC amplitude was not altered at 24 h post-OGD (Fig. 10*G*; 30.34 pA ± 1.403 *versus* 30.05 pA ± 1.231, two-tailed unpaired *t* test *p* = 0.88). Similarly, consistent with the morphological reduction in gephyrin cluster density, interevent intervals of GABAergic IPSCs were also significantly increased at 24 h after OGD (Fig. 10*H*; 198.2 ms ± 13.34 *versus* 146.0 ms ± 10.25, two-tailed unpaired *t* test *p* = 0.0046). Importantly, scavenging BDNF using TrkB-Fc (1 mg/ml) prevented the loss of GABAergic inhibition at 24 h post-OGD. Analysis showed that TrkB-Fc application prior to OGD does not influence the mIPSC amplitude (Fig. 10*G*, 28.63 pA ± 3.02 *versus* 28.77 pA ± 2.6, two-tailed unpaired *t* test *p* = 0.97). Similarly, the loss of GABAergic synapses was prevented in slices treated with TrkB-Fc prior to OGD (Fig. 9*H*, 182.8 ms ± 19.25 *versus* 141.9 ms ± 15.49, two-tailed unpaired *t* test *p* = 0.114). Taken together, our data establish a direct link between BDNF signaling at 24 h post-OGD with morphological changes in gephyrin clustering and functional alteration in GABAergic transmission.

In primary hippocampal neurons, we demonstrate that gephyrin SUMO-defective mutants K148R and K724R are nonresponsive to BDNF treatment (Fig. 2, *D* and *E*). We wondered whether SUMOylation-defective gephyrin mutant transgene expression in CA1 hippocampal slices could also prevent gephyrin cluster loss 90 min after OGD. To test this idea, we cotransfected organotypic hippocampal culture CA1 pyramidal neuron with TdTomato and eGFP-gephyrin or eGFP-K148R/K724R gephyrin SUMO-1- and SUMO-2/3-defective combination mutant using biolistic gene gun. Morphological analysis for changes in eGFP-gephyrin cluster size and density at 90 min post-OGD showed significant reduction in cluster size (Fig. 10,*I* and *K*; 0.03 μm^3^ ± 0.002 *versus* 0.019 μm^3^ ± 0.0018, KS test T < 0.001) and reduced density of gephyrin clusters (Fig. 10*L*; 0.65 ± 0.01/μm *versus* 0.33 ± 0.03/μm; two-tailed unpaired *t* test *p* = 0.001). Interestingly, transgenic expression of gephyrin SUMO-defective K148R/K724R mutant could rescue the reduction in gephyrin cluster volume after OGD (Fig. 10*K*; 0.03 μm^3^ ± 0.005 *versus* 0.046 μm^3^ ± 0.003, KS test *p* = 0.116), and prevent the reduction of gephyrin cluster density (Fig. 10*K*; 0.75 ± 0.15 *versus* 0.82 ± 0.07; two-tailed unpaired *t* test *p* = 0.93). Our results show that gephyrin is a direct molecular substrate after OGD to influence GABAergic inhibition. Furthermore, we identify a previously unknown link connecting BDNF signaling to the SUMO pathway proteins, specifically PIAS-3 to influence GABAergic inhibition after OGD.

## Discussion

In the present study, we demonstrate that BDNF signaling shuttles SUMO-1 and SUMO-2/3 from the nucleus into the soma and dendrites in a time-dependent manner. The TrkB receptor downstream of BDNF activates ERK1/2 pathway to impinge upon gephyrin and PIAS-3, influence their cooperativity, and in turn impact GABAergic inhibition. PIAS-3 and gephyrin exhibit more than one biochemical interaction site, which allows for PIAS-3 to influence gephyrin clustering *via* its RING and S/T domains. This influence of PIAS-3 on gephyrin clustering is in turn regulated by ERK1/2 kinase pathway and phosphorylation at Ser268 residue on gephyrin. Using OGD as *in vitro* model for brain ischemia, we demonstrate that after OGD, there is increased BDNF mRNA. Using TrkB-Fc chimera to sequester BDNF signaling in our OGD model, we could prevent reduction of gephyrin cluster density and downregulation in GABAergic inhibition. At 24 h post-OGD, BDNF signaling *via* TrkB receptor and downstream ERK1/2 pathway converge on PIAS-3 and gephyrin to influence functional adaptation at GABAergic postsynaptic sites. We report that kinase and SUMO pathways converge on determining the outcome of BDNF signaling and PIAS-3 function. Specifically, gephyrin phosphorylation by ERK1/2 on S268 and SUMO-1/-2 conjugation on K148R/K724R renders gephyrin insensitive to PIAS-3. Our data highlight that in physiology and pathology, cellular signaling cascades crosstalk with each other to influence gephyrin posttranslational modification(s) and in turn impact GABAergic inhibitory neurotransmission.

### BDNF signals for PIAS-3 and gephyrin cooperativity

Our biochemical analysis identified more than one interaction site for gephyrin on PIAS-3 and vice versa. It has been reported that gephyrin is SUMO-1 conjugated at the K148 (G domain) and SUMO-2 conjugated at K724 (E domain) residues (13). The identification of PIAS-3 binding site(s) on gephyrin indicates that this could be the basis for gephyrin SUMO conjugation. It has been reported in stem cells that PINIT domain mutation leads to both nuclear and cytosolic localization of PIAS-3 (35).

As a proof of principle, we demonstrate that gephyrin SUMO-1 and SUMO-2/3 site mutations K148R and K724R, respectively, are insensitive to BDNF signaling (Fig. 2). Several neuronal proteins have been characterized as novel SUMO1 substrate *in vivo* (36); however, there is little mechanistic understanding of how SUMOylation is achieved at synaptic locations. Our data offer an elegant model for nucleo-dendritic shuttling of SUMO1/2/3 and PIAS-3 in response to BDNF signaling, thereby facilitating SUMOylation of synaptic proteins. We also provide evidence showing long-term BDNF treatment (48 h) renders proteins of the SUMO pathway insensitive to BDNF, again causing these proteins to relocalize within the nucleus. It is well accepted in the field that protein SUMOylation is a labile process; however, within the neuronal context, our data offer a mechanistic underpinnings of a dynamic regulatory process.

Our results show that myc-PIAS-3, myc-PIAS-3Rm, and myc-PIAS-3 S/T domains restore gephyrin cluster size and density to base line condition upon blocking of ERK1/2 signaling. We show that BDNF treatment restores gephyrin cluster size but not density in myc-PIAS-3–overexpressing neurons (Fig. 7*A*). However, PD98059 treatment restores both cluster size and density in myc-PIAS-3–overexpressing cells (Fig. 4*A*). Importantly, PD98059 treatment restores cluster size and density in neurons overexpressing the PIAS3Rm or S/T domain (Fig. 9, *A*–*F*). We envision a scenario wherein RING domain and S/T domain control the regulation of gephyrin size and density, respectively. Given that ERK1/2 also phosphorylates gephyrin at S268 to reduce cluster size (13), BDNF treatment could reduce the gephyrin cluster size *via* this direct phosphorylation event. However, in parallel, BDNF activates PIAS-3 to influence its SUMOylation function. Hence, PIAS-3 effect on gephyrin clustering occurs downstream of ERK1/2 pathway involving the PIAS-3 Ring and S/T domains *via* mechanisms that we do not understand fully.

### BDNF signaling and gephyrin modulation for brain network integrity

Our observations confirm that BDNF and not NT-4 *via* TrkB receptor activates ERK1/2 pathway downstream to influence PIAS-3 function and gephyrin SUMOylation. This is consistent with established literature showing BDNF–TrkB interaction but not NT-4–TrkB interaction leads to less efficient sorting of TrkB receptors and enhanced activation of downstream signaling (23). The signaling downstream of BDNF is mediated by the Shc adaptor binding site on TrkB and Ras/MAPK pathway activation. The generation of mouse line harboring the Shc binding site mutation in the *trkB* gene has helped to delineate that NT4-dependent signaling is independent of BDNF-dependent signaling. Also, neurons derived from *trkB*^shc/shc^ mutant mice do not show any defects in BDNF-dependent signaling (24). Our results are consistent with these reports and show that BDNF and not NT-4 signaling through TrkB receptor regulates GABAergic synapse plasticity. We report that dynamic time scale of synaptic plasticity adaptations is facilitated by ERK1/2 pathway directly impinging on PIAS-3 localization and function. PIAS-3 function for gene transcription regulation in photoreceptor cells has been reported (6). Our data provide a molecular framework for PIAS-3 function at synaptic sites.

Dynamic modulation of GABAergic inhibition is especially relevant within the context of synaptic homeostasis, wherein individual neurons and/or synapse adapts to fluctuations in activity. In addition, sensory input–dependent adaptations in GABAergic inhibition and gephyrin clustering have been reported (37, 38, 39). Furthermore, during a narrow postischemic timeframe, synaptic plasticity plays an important role in the recovery process (40). Posttranslational modification like SUMOylation of cellular proteins are thought to contribute to the recovery process after ischemic insult (16). Although, elevated SUMO-conjugated proteins and BDNF levels after an ischemic stroke have been reported in literature (18), a functional link between BDNF and SUMO pathway has not been reported so far. Our study provides the first evidence linking BDNF signaling with the regulation of SUMO pathway.

## Experimental procedures

All animal experiments were approved by the cantonal veterinary office of Zurich (ZH011/19). All experiments were performed in accordance with guidelines from the Swiss Veterinary office or Canadian Council on Animal Care and the National Institutes of Health in the USA. All animal procedures at McGill were approved by the Animal Resource Committee of the School of Medicine at McGill University Protocol number 5057.

### Plasmids

EF1a-eGFPC2-gephyrin has been described earlier (41); eGFPC2-S268E has been described earlier (26); pCMV ± 6xmyc (PIAS1, PIAS2α, PIAS2β, PIASγ) has been described earlier (31). pCMV ± 6xmyc-PIAS-3 (1-584AA and C299S/H301A SP-Ring mutant) has been described earlier (31); eGFP-PIAS-3 was a kind gift from Prof. Johar Yogil (Hebrew University, Jerusalem). The plasmids pCMV ± 6xmyc–PIAS-3 (1-273AA, 274-392AA, 274-584AA, 393-584AA and 416-584AA) were kind a gift from Prof. Fang (Rutgers University, New Jersey, USA). pCMV ± 6xmyc-PIAS-3(1-406) was generated by deleting the C-terminus domain from pCMV ± 6xmyc-PIAS-3. eGFP-gephyrin SUMO-1 and SUMO-2 site mutations (K148R and K724R) are described in (13).

### Primary hippocampal neuronal culture

Dissociated embryonic (E17-E18) Wistar-rat hippocampal primary mixed cultures were prepared as described earlier (41). They were maintained in the culture media containing MEM (Gibco), 15% Nu-serum (Becton-Dickinson,355500), B27 supplement (Invitrogen), 1 M Hepes (pH7.2; 15 mM), glucose monohydrate (0.45%), 1 mM Na-pyruvate, and 2 mM L-Glutamine. The cells were transfected following the protocol described in T. Buerli *et al*. 2007 (42) at DIV 8 with 1 μg total plasmids DNA with up to a total of three different plasmids transfected simultaneously. We used Lipofectamine 2000 (Invitrogen, 11668-019), CombiMag (Oz Biosciences, CM21000), and OptiMEM medium (Invitrogen, 31985-070) as per the protocol.

### Immunohistochemistry of primary cells culture

Seven days posttransfection, the cells (8 + 7DIV) were fixed in 4% paraformaldehyde for 10 min, then permeabilized for 5 min with 0.1% Triton X-100 in 10% normal goat serum (NGS, Bio-Rad, C07SA) and PBS, pH 7.4. The cells were quickly washed with PBS (pH7.4) before being labeled with the appropriate primary antibody cocktail (antibodies with 10%NGS and PBS) for 90 min. After three washes of 10 min each with PBS, the secondary detection was achieved with the secondary antibody mixture supplemented with DAPI (1:1000) for 30 min. The coverslips were mounted with Dako Fluorescence Mounting medium (Dako North America, Inc).

### Antibodies

Mouse anti-Gephyrin (1:1000, clones mAb7a, Synaptic Systems #147021), rabbit anti-SUMO-1 (1:250, Epitomics#1563-1), mouse anti-SUMO-1 (1:100, SantaCruz#sc-5308), rabbit anti-SUMO-2/3 (1:250, Cell signaling #4974), rabbit anti-SUMO-2/3 (1:250, Epitomics #2970-1), mouse anti-PIAS-3 (1:500, Sigma #P0117), rabbit anti-vGAT (1:2000, Synaptic Systems #131011); mouse anti-Myc tag (1:5000,Roche #11667149001), rabbit anti-Myc tag (1:5000, Cell Signaling #2278S), and mouse anti-FLAG tag (1:5000, Sigma Aldrich #F3165). All the secondary antibodies were from Jackson ImmunoResearch: Goat anti-Mouse Cy3 IgG (1:500, #115165), Goat anti-Mouse IgG Cy5 (1:500, #115175), Goat anti-Rabbit IgG Cy3 (1:500, #111165), and Goat anti-Rabbit IgG Cy5 (1:500, #111175).

### Pharmacological treatments

Transfected cells were treated 90 min with hBDNF (10 ng/ml, Alomone Labs #B-250), NT-3 (10 ng/ml, Alomone Labs #N-260), or NT-4 (10 ng/ml, Alomone Labs #N-270) and/or rh TrKB-Fc (1 μg/ml, R&D Systems #688-TK-100). Otherwise, the cells were treated overnight with ERK 1/2 inhibitor: PD98059 (25 μM/ml, Calbiochem#513000) or GSK3β inhibitor: GSK3βIX (5 μM/ml, Calbiochem #328007) or DMSO (equal volume; Sigma D2438) & pharmacological inhibitor (ANA-12, 400 nM, Sigma-Aldrich #SML0209).

### Image analysis and quantification

All images were acquired on confocal laser scanning microscope (LSM 710, Carl Zeiss) with objective lens of 40× (NA 1.4) with a pinhole set at 1 Airy unit and a pixel size of 0.13 μm. For each condition, images from a minimum of 9 cells from three independent batches of neuronal culture were acquired using a z-stack (3–5 steps at 0.5 μm per step size). From each cell, a dendritic segment was taken for analysis. Image analyses were performed with a custom written analysis for Image J software using maximal intensity z-projected images.

Gephyrin clustering size area and density were analyzed 7 days posttransfection in hippocampal primary neuronal culture following the protocol previously described (43, 44). The generated data are then plotted using Excel software and GraphPad Prism software.

### Statistical analysis

When multiple groups were compared using either two-way ANOVA or one-way ANOVA followed by a Bonferroni pair-wise comparison as indicated and Mann–Whitney pair-wise comparison as indicated.

### HEK 293 cell cultures and transfection

Human embryonic kidney (HEK293) cells were maintained at 37 °C and 5% CO_2_ in Dulbecco’s modified Eagle’s medium (Gibco 41966-029), supplemented with 10% fetal calf serum (Gibco #10270-106). They were transfected, 24 h postplating, with either 1 μg (for all gephyrin constructs) or 2 μg (for all PIAS-3 constructs) of DNA using poly-ethylamine (Polysciences Inc, 23966) according to the manufacturer recommendation. Twenty-four hours later, the cells were lysed in EBC buffer (50 mM Tris pH 8.0, 120 mM NaCl, 0.5% NP-40) containing proteasome inhibitor or complete-mini protease inhibitor cocktail tablets (Roche diagnostic, #11836153001) and phosphatase cocktail 2 and 3 (Sigma #P5726 and #P0044).

### Immunoprecipitation and Western blot

Interaction between two proteins was determined using the heterologous cells HEK293. For the IP followed by Western blot (WB) assays, the cell lysates were incubated 90 min at 4 °C with 1 to 2 μg purified antibody followed by incubation with protein A/G UltraLink Resin (Thermo Scientific, #53133) 45 min at 4 °C. Unspecific binding to the resin was minimized by washing with EBC-based high-salt buffer (50 mM Tris, 500 mM NaCl, 1% NP-40) followed by washes with normal EBC buffer. The samples were boiled with SDS sample buffer containing 15% fresh β-mercaptoethanol at 90 °C for 4 min and separated on appropriate acrylamide % SDS gel at 140 V. The proteins were transferred onto a PVDF membrane on which the WB could be performed. The membrane was blocked with 5% Western blocking reagent (Roche, #11921681001), then incubated with the primary antibody mixture for 3 h or overnight. After washing with Tris-buffered saline with Tween20 (TBS-T), the membranes were incubated with the secondary antibodies mixture containing either Donkey horse radish peroxidase antibodies (HRP 1:10,000, form Jackson ImmunoResearch: mouse #715-035-150 and rabbit #711-035-152) or fluorescent secondary’s (1:30,000): mouse IR680 (#926-68022) or rabbit IR 800 (#926-32213) from Odyssey-AB/Li-COR. For loading controls, protein lysates were boiled with 5*×* SDS buffer before performing WB with the appropriate antibodies.

### Whole cell patch clamp recording in primary neuron

Whole cell voltage clamp recordings were performed in rat hippocampal cultured neurons (11 + 3 DIV) at RT. Thick-walled electrodes were pulled from a borosilicate glass pipette (30-0057, Warner instruments) to 3 to 5 MΩ using a vertical electrode puller (PC-100, NARISHIGE Group). Recording pipette was filled with Cesium-based internal solution containing (in mM): 120 CsCl, 10 EGTA, 10 Hepes pH 7.4, 4 MgCl2, 0.5 GTP, and 2 ATP. The external solution (7.4 pH, 310 mOsm) contained (in mM): 125 NaCl, 2.5 KCl, 1.25 NaH2PO4, 26 NaHCO3, 25 D-glucose, 2.5 CaCl2, and 2 MgCl2.

Miniature inhibitory postsynaptic currents (mIPSCs) were isolated by adding 6-cyano-7-nitroquinoxaline-2,3-dione (CNQX) (25 μM, Merck), AP-5 (50 μM, Alomone Labs), and tetrodotoxin (1 μM, Affix Scientific). Cell were recorded at holding potential of −70 mV. Recordings were amplified by Multiclamp 700B amplifier and digitized with Digidata 1440 (Molecular Devices). Cells were recorded for a total duration of 5 min: after 3 min of establishing a stable whole-cell mode, mIPSCs were analyzed for the last 2 min. Only cells which showed a stable recording in the first 3 min, series resistance increase <30%, and signal above the noise background (5–10pA) were further analyzed. The decay time of mIPSCs was fitted with a single exponential curve and fitted between 10 and 90% of its amplitude. Events were recorded using Clampex 10.7 software (Molecular Devices) with sampling rate of 10 kHz and filtered offline using Bessel low pass filter (Clampfit 10.7) and analyzed using MiniAnalysis 6.0.7 (Synaptosoft).

### Whole cell patch clamp recording in organotypic hippocampal slice culture

All electrophysiological recordings were made using an Axopatch 200A amplifier (Molecular Devices). GABA_A_R-mediated mIPSCs were gathered from whole-cell voltage-clamp recordings of CA1 pyramidal neurons obtained at 25 °C using electrodes with resistances of 4 to 5 MΩ and filled with intracellular solution containing (in mM): CsCl, 140; NaCl, 4; 0.5, CaCl2; Hepes, 10; EGTA, 5; QX-314, 2; Mg-ATP, 2; Na-GTP, 0.5; and 290 mOsm, pH adjusted with CsOH to 7.36. mIPSCs were recorded at −60 mV and in the presence of 1 μM tetrodotoxin, 25 μM CPP, 5 μM CGP55845, 5 μm 6-cyano-7-nitroquinoxaline-2,3-dione (CNQX), and 0.3 μm strychnine in external Tyrode’s solution. Access resistance was monitored with brief test pulses at regular intervals (2–3 min) throughout the experiment. After the holding current had stabilized, data were recorded at a sampling frequency of 10 kHz and filtered at 2 kHz for 10 to 15 min. mIPSCs were detected offline using the Mini Analysis Software (Synaptosoft). The amplitude threshold for mIPSCs detection was set at four times the root-mean-square value of a visually event-free recording period. From every experiment, 5 min of stable recording was randomly selected for blinded analysis of amplitude and interevent interval. The data obtained were then used to plot cumulative histograms with an equal contribution from every cell.

### Organotypic hippocampal slice cultures

Organotypic hippocampal slices (400 μm thickness) were obtained from postnatal day 7 C57BL/6J mice or transgenic mice expressing MARCKs-enhanced GFP tagged to the CA1 neuronal membrane. Tissue slices of 400 μm thickness were prepared following the roller-tube method from Gähwiler technique (45). The slices were incubated in an antibiotic-free serum medium containing 25% heat-inactivated horse serum, 25% Hank’s balanced salt solution, and 50% Basal Medium Eagle. They were maintained for 3 weeks minimum allowing maturation prior to experimentation at 36 °C in a roller drum incubator.

Images were acquired on a Leica DM6000B laser scanning microscope (Leica Microsystems) with an objective lens of 63*×* NA 1.4 oil immersion. At least three slices from three independent batches per condition were acquired (0.3 μm z stack). Image analysis of gephyrin clustering in the hippocampal CA1 region were done, postdeconvolution with Huygens Essential software, using the Surpass and the Spot functions of Imaris 7.00 software (Biplane AG).

### Organotypic slice transfection

eGFP-gephyrin, eGFP-K148R, or eGFP-K724R and pCR3-Td-Tomato were cotransfected into DIV 14 organotypic slices using the Helios Gene Gun (Bio-Rad laboratories), following the vendor protocol.

### OGD treatment

The slices were incubated in glucose-free Tyrode (ACSF) solution supplemented with 2 mM 2-deoxyglucose, 8 mM sucrose, and 3 mM sodium azide (NaN_3_) and bubbled with 95%N_2_/5%CO_2_. The slices were incubated during 4 min in the OGD solution or normal Tyrode solution (control conditions) and returned in normal culture medium for 90 min, 24 h before experimenting as a model for ischemic injury *in vitro* (46).

### Immunohistochemistry of organotypic hippocampal slice cultures

Slices were fixed using 4% paraformaldehyde for 1 h and washed with 0.1 M phosphate buffer, subsequently permeabilized using 0.4% Triton x100 and blocked with 1.5% heat-inactivated horse serum overnight at 4 °C. The primary antibody cocktail were incubated (in permeabilizing buffer) over 5 days at 4 °C. The slices were then washed several times with 0.1 M PBS during the whole day, followed by the incubation with the secondary antibody mixture overnight at 4 °C. Slices were mounted using Dako Fluorescence Mounting medium (Dako Canada).

### Real-time qPCR

Areas CA1 and CA3 were microdissected from five to six slices from three independent litter and used for each experimental condition. Total mRNA was extracted using BioRad extraction kit. Subsequently, 1 μg of mRNA was reverse transcribed to cDNA following the manufacturer’s protocol (Roche Diagnostic). The RT-qPCR was performed using 30 ng of cDNA in a 20 μl reaction mixture containing EVA green mastermix (Solis BioDyne #08-24-00008). All qPCR reactions were performed under those conditions: 40 cycles; denaturation at 95 °C for 15 s, annealing at 62 °C for 25 s, and extension at 72 °C. Primers: the following primer pairs were used for each reaction: *bdnf* Fwd: 5′-TGC AGG GGC ATA GAC AAA AGG-3′, Rev: 5′-CTT ATG AAT CGC CAG CCA ATT CTC-3’; *Gapdh* Fwd: 5′-TGCCCCCATGTTTGTGATG-3′ Rev: 5′-TGTGGTCATCAGCCCTTCC-3'.

## Data availability

The authors declare no restrictions on data availability.

## Supporting information

This article contains supporting information.

## Conflict of interest

The authors declare that they have no conflicts of interest with the contents of this article.

## Supporting information

Fig.S1 **Subcellular localization of PIAS-3 is specifically influenced by BDNF.***A*, morphology of transfected neuron showing eGFP-gephyrin and myc-PIAS-1, myc-PIAS2, myc-PIAS3, and myc-PIASγ treated with BDNF (90 min). The myc (*red*) shows that all the PIAS except PIAS3 change subcellular localization after BDNF application. Scale bar 5 μm. BDNF, brain-derived neurotrophic factor; PIAS, protein inhibitor of activated STAT.

Fig. S2 **Gephyrin ERK mutant impairs PIAS-3 induced clustering changes**. *A–B*, morphology of dendrite segement cotransfected with eGFP-gephyrin or eGFP-S268E mutant and myc-PIAS-3. Quantification of eGFP-gephyrin cluster size and density in eGFP-S268E and myc-PIAS3 expressing neurons show significant reduction in size but not density. *C*–*D*, morphology of dendrite segement cotransfected with eGFP-gephyrin or eGFP-S268E mutant and myc-PIAS-3Rm. Quantification of eGFP-gephyrin cluster size and density in eGFP-S268E and myc-PIAS3Rm expressing neurons show significant reduction in size and density. *E*–*F*, morphology of dendrite segement cotransfected with eGFP-gephyrin or eGFP-S268E mutant and myc-RING. Quantification of eGFP-gephyrin cluster size and density in eGFP-S268E and myc-RING expressing neurons show significant reduction in size and density. *G*–*H*, morphology of dendrite segement co-transfected with eGFP-gephyrin or eGFP-S268E mutant and myc-S/T. Quantification of eGFP-gephyrin cluster size and density in eGFP-S268E and myc-S/T expressing neurons show significant reduction in size but not density. Data were collected from three independent experiments. Two-tailed Student *t* test. Error bars st.dev. Scale bar 10 μm. ∗p < 0.05 and ∗∗∗p < 0.0001. PIAS, protein inhibitor of activated STAT
