# Supplementary figures and images for: Trophic factor BDNF inhibits GABAergic signaling by facilitating dendritic enrichment of SUMO E3 ligase PIAS3 and altering gephyrin scaffold
Source: J Biol Chem. 2022 Mar 17;298(5):101840. doi: 10.1016/j.jbc.2022.101840 (PMC9019257; doi:10.1016/j.jbc.2022.101840)

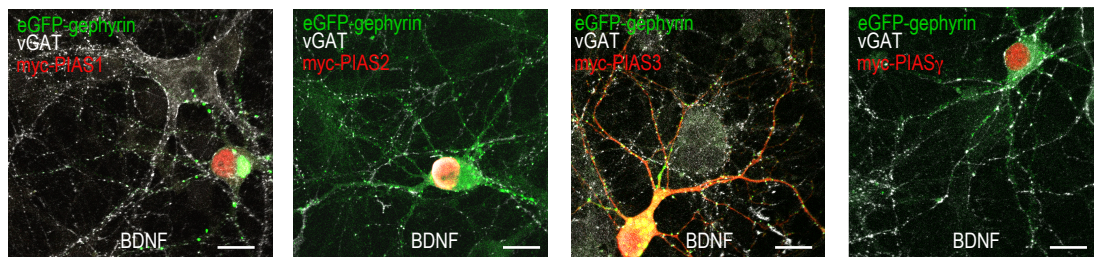

Thirouin et al., Suppl. Fig. 1

Supplement: Fig.S1 — Subcellular localization of PIAS-3 is specifically influenced by BDNF.A, morphology of transfected neuron showing eGFP-gephyrin and myc-PIAS-1, myc-PIAS2, myc-PIAS3, and myc-PIASγ treated with BDNF (90 min). The myc (red) shows that all the PIAS except PIAS3 change subcellular localization after BDNF application. Scale bar 5 μm. BDNF, brain-derived neurotrophic factor; PIAS, protein inhibitor of activated STAT. [file mmc1.pdf]

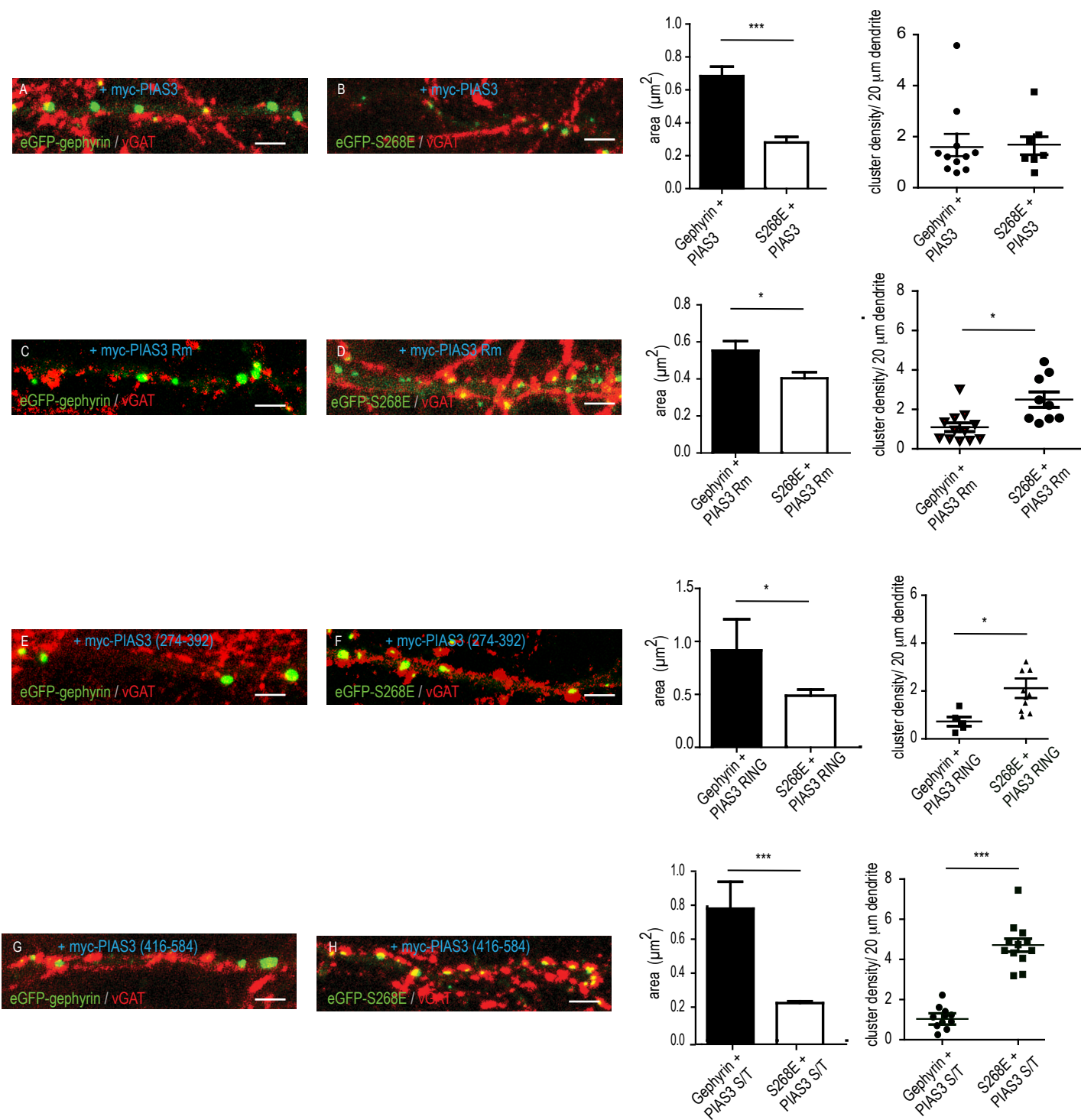

Supplement: Fig. S2 — Gephyrin ERK mutant impairs PIAS-3 induced clustering changes. A–B, morphology of dendrite segement cotransfected with eGFP-gephyrin or eGFP-S268E mutant and myc-PIAS-3. Quantification of eGFP-gephyrin cluster size and density in eGFP-S268E and myc-PIAS3 expressing neurons show significant reduction in size but not density. C–D, morphology of dendrite segement cotransfected with eGFP-gephyrin or eGFP-S268E mutant and myc-PIAS-3Rm. Quantification of eGFP-gephyrin cluster size and density in eGFP-S268E and myc-PIAS3Rm expressing neurons show significant reduction in size and density. E–F, morphology of dendrite segement cotransfected with eGFP-gephyrin or eGFP-S268E mutant and myc-RING. Quantification of eGFP-gephyrin cluster size and density in eGFP-S268E and myc-RING expressing neurons show significant reduction in size and density. G–H, morphology of dendrite segement co-transfected with eGFP-gephyrin or eGFP-S268E mutant and myc-S/T. Quantification of eGFP-gephyrin cluster size and density in eGFP-S268E and myc-S/T expressing neurons show significant reduction in size but not density. Data were collected from three independent experiments. Two-tailed Student t test. Error bars st.dev. Scale bar 10 μm. ∗p < 0.05 and ∗∗∗p < 0.0001. PIAS, protein inhibitor of activated STAT [file mmc2.pdf]
